# Robustness and Sensitivity of Gd(III)–Gd(III) Double Electron–Electron Resonance (DEER) Measurements: Comparative Study of High-Frequency EPR Spectrometer Designs and Spin Label Variants

**DOI:** 10.1007/s00723-024-01741-0

**Published:** 2025-01-03

**Authors:** Elena M. Mocanu, Yasmin Ben-Ishay, Lydia Topping, S. Ronan Fisher, Robert I. Hunter, Xun-Cheng Su, Stephen J. Butler, Graham M. Smith, Daniella Goldfarb, Janet E. Lovett

**Affiliations:** 1 SUPA School of Physics and Astronomy and BSRC, University of St Andrews, North Haugh, St Andrews, KY16 9SS UK; 2 Department of Chemical and Biological Physics, Weizmann Institute of Science, 7610001 Rehovot, Israel; 3 Department of Chemistry, Loughborough University, Epinal Way, Loughborough, LE11 3TU UK; 4 State Key Laboratory of Elemento-Organic Chemistry, Tianjin Key Laboratory of Biosensing and Molecular Recognition, College of Chemistry, Nankai University, Tianjin, China

## Abstract

In this paper, we explore the robustness and sensitivity of Gd(III)-Gd(III) double electron–electron resonance (DEER) distance measurements in proteins for different spectrometer designs and three spin labels. To do this a protein was labeled at the same two positions with Gd(III) spin labels and measurements were performed on two home-built high-frequency (W-band, ~ 95 GHz) EPR spectrometers with different design approaches, and a commercial 150 W Q-band (34 GHz) spectrometer. The first W-band measurement approach uses a conventional, narrow band single mode cavity, while the second uses a broadband non-resonant induction mode sample holder. Both systems incorporate advanced arbitrary waveform generators (AWGs) that give flexibility over excitation bandwidth. We use three DOTA-like Gd(III) spin labels, Gd.C12, Gd.DO3A and Gd.L^1^, conjugated to the calmodulin protein. We compare measurements taken by including or excluding the Gd(III) central transition excitation. The advantages and disadvantages of the EPR spectrometers for the measurement of Gd(III)–Gd(III) DEER are discussed in terms of the robustness of the resulting distance distribution width, absolute and concentration sensitivity, sample handling, ease of use, and flexibility of measurement.

**Supplementary Information:**

The online version contains supplementary material available at 10.1007/s00723-024-01741-0.

## Introduction

**Fig. 1:**
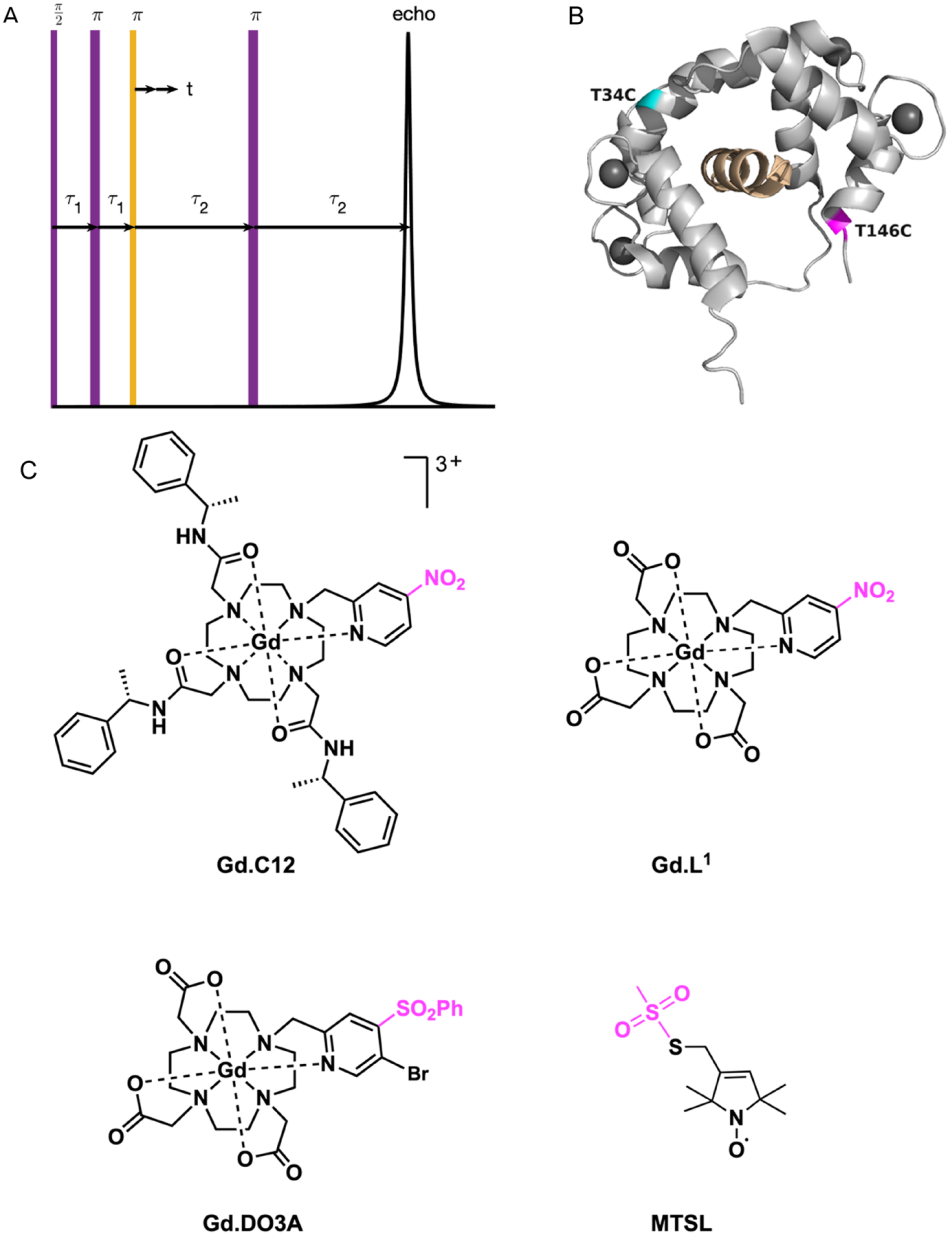
The DEER pulse sequence, labels and protein used. **A** The four-pulsed DEER sequence. The observer frequency refocussed echo sequence is represented in purple and the pump pulse at a second frequency is in orange. The pulses are represented as rectangular for convenience but take various forms in this work. **B** The calmodulin (CaM) protein NMR structure (2BBM.pdb [42]) in grey with dark grey spheres representing the four bound Ca(II) ions and the M13 peptide in gold. In this work, the CaMM13 is a single amino acid chain with M13 attached through a GG linker to the C-terminal of CaM. The two genetically engineered cysteines used for spin labeling are highlighted in cyan (T34C) and magenta (T146C). **C** The four spin labels used in this work. The group that leaves through the conjugation of the label to a cysteine residue is highlighted in magenta

Gadolinium(III) ion (Gd(III)) complexes are high-spin ${\text{S}} = 7/2$ paramagnetic centres that have been developed as spin label probes for pulse dipolar EPR spectroscopy [1–4]. The pulsed dipolar measurements, such as those acquired by double electron–electron resonance (DEER), are then analyzed to determine nanometer-scale distances and distance distributions, Fig. 1A [5–13]. These data are crucial for the structural determination of proteins both in-vitro and in-cell, where quinary structure may be present inside the protein’s native cell [14–31]. The advantages of Gd(III) labels for in-cell measurements, compared to nitroxide labels, include high chemical stability in the reductive cellular milieu and exceptional concentration sensitivity at high magnetic fields, owing to their high spin and spectroscopic properties [28].

In the high field limit and frozen solutions, the Gd(III) spin label complexes have an EPR spectrum that consists of a sharp (intense) central line from the ${\text{m}}_{\text{s}} = - 1/2$ to ${\text{m}}_{\text{s}} = + 1/2$ transition and a broad, usually featureless, background arising from all other transitions [32]. The breadth of the central transition is proportional to ${D}^{2}/{{\nu}_{0}}$, where *D* is the zero-field splitting (ZFS) parameter and ν_0_ is the EPR spectrometer (Larmor) frequency. As the EPR frequency increases the width of the Gd(III) central transition line decreases, leading to increased EPR-detection sensitivity. Since the absolute sensitivity of the DEER experiment is a product of the detected echo intensity from the Gd(III) ion and the modulation depth, it would appear advantageous for the DEER experiment to utilize a Gd(III) spin label with a narrow central transition [2, 18, 33, 34]. This may be by using high field (most often W-band ~ 95 GHz) or a Gd(III) label with a small ZFS, or a combination of both factors. Usually, the DEER pump pulse frequency matches the central transition to increase the modulation depth since many spins will be inverted. However, a narrow central transition indicates a small ZFS, and in this regime, the pseudo-secular mixing terms in the dipolar Hamiltonian must be taken into account if both the observed and pump frequencies affect either the ${\text{m}}_{\text{s}} = - 1/2$ or ${\text{m}}_{\text{s}} = + 1/2$ levels [10, 11, 13]. In many spectrometers, this is a situation that is difficult to avoid because of bandwidth limitations, and therefore distance measurements less than around 3 nm (for most Gd(III) spin labels) may be artificially strongly broadened. This can be avoided by increasing the frequency separation between the pump and observer pulses such only one of them affects either the ${\text{m}}_{\text{s}} = - 1/2$ or ${\text{m}}_{\text{s}} = + 1/2$ level [35]. It has also been shown that completely avoiding the central transition while maintaining a large frequency separation between the observer and pump frequencies can eliminate this broadening with little or no loss of signal-to-noise ratio [13]. Those measurements were taken using rectangular pulses, but today arbitrary waveform generators, AWGs, which enable shaped and chirped pulses, allow increased fidelity, selectivity and excitation bandwidth [36].

The aim of this paper is to explore the robustness and sensitivity of Gd(III)-Gd(III) DEER distance measurements across different spectrometers, spin labels, and different measurement approaches. We compared the experimental measurement procedures and the consistency of the results for Gd(III) DEER experiments using two home-built W-band spectrometers that are located at the Weizmann Institute of Science (WIS) in Israel and the University of St Andrews in the UK (the HiPER spectrometer). They differ considerably in their design but are both state-of-the-art. HiPER uses a non-resonant sample holder with a flat frequency response in conjunction with an EIK amplifier to provide up to 1.3 kW pulse power over the EIKA’s nominal 1 GHz bandwidth. It is controlled by an AWG that allows both amplitude and phase control of pulses over the 1 GHz bandwidth [37]. This enables wideband excitation outside of the central transition. The main features of the WIS spectrometer are that it also uses an AWG, has a narrow band cylindrical cavity and the W-band bridge maximal output power is 3 W (Quinstar QPP-95013530-H8W04) [36, 38–40]. Further information on both spectrometers is available in the supporting information.

The relative advantages, and disadvantages, of each design have not been previously compared: for example, while HiPER may offer greater concentration sensitivity, and the WIS spectrometer greater absolute sensitivity based on their design specifications, the degree to which this is true and the subtleties of how that affects the measurement of Gd(III)-spin-labeled proteins have not, until now, been assessed. The W-band data are further compared with those acquired on a 150 W Q-band Bruker spectrometer (ER 5106QT-2w cylindrical resonator), located at the University of St Andrews. It is included in this study to compare the homebuilt spectrometer performances for measuring Gd(III) DEER, to the much more readily available commercial Q-band system. To achieve the aims stated above, a protein with an expected inter-label distance of around 4 nm was doubly spin labeled with three DOTA-like Gd(III) spin labels. Therefore, this work also provides a comparison of the methodological use and performance of DEER measurements of these three seemingly similar spin labels.

The protein chosen for this work is a hybrid of recombinant human calmodulin (CaM) with a target binding peptide (M13) fused to its C-terminal (CaMM13) [41]. In the presence of calcium ions, the protein folds around the M13 and forms a compact structure with the spin labels predicted to be solvent-exposed. The 2BBM.pdb NMR-derived structure of CaM with M13 peptide is shown in Fig. 1B, with positions 34 and 146 highlighted [42]. These amino acids were mutated to cysteines for spin labeling. The double cysteine variant has been previously measured (in hitherto unpublished work) with the nitroxide spin label MTSL (1-oxyl-2,2,5,5-tetramethyl pyrroline-3-methyl)methanethiosulfonate) (Fig. 1C). For comparison, this DEER data measured at Q-band will be presented alongside the results using Gd(III) spin labels at W- and Q-bands.

Gd(III) spin labels are typically based on kinetically stable macrocyclic Gd(III) complexes which can be readily modified for particular chemical or environmental requirements. In this study, we used three Gd(III) spin labels to enable comparison of the spectrometers over several samples to assess robustness. The use of three labels conveniently also allows us to investigate the comparative chemical reactivity and conformational freedom of the spin labels. Based on previous studies, the labels are all expected to have a moderate ZFS i.e. with a zero field *D* parameter in the range 700 to 1200 MHz [16, 43, 44]. The three labels are based on a common macrocyclic scaffold that provides octadentate coordination to the central Gd(III) ion, via nitrogen and oxygen donor atoms, including one nitrogen from a pyridine pendant arm (Fig. 1C). A water molecule or hydroxyl ion presumably fills the ninth coordination site in an aqueous solution. The pyridine arm in each label is further modified to allow for covalent bonding to a cysteine sulfhydryl group, forming a stable thioether bond. All labels have the same tether to the protein and are therefore expected to have similar rigid conjugation to the protein.

Gd.DO3A is an overall neutral Gd(III) complex that has been utilized in several DEER studies due to its ease of labeling and favorable conformational degrees of freedom for reliably reproducible and relatively small distance distributions once tagged onto a protein [23, 25, 31, 45]. Upon conjugation with a cysteine residue, the –SOPh_2_ group is displaced, with the ortho bromide group affording extra chemical reactivity.

Complex Gd.L^1^ is a structurally related and also overall neutral complex, bearing a 4-nitropyridine arm with an -NO_2_ leaving group, which also provides a thioether bond to the protein. The attached spin label is almost identical to Gd.DO3A-protein, except for the presence of the pyridyl bromine atom in the latter. While the homologous complexes Eu.L^1^ and Dy.L^1^ have been previously reported for protein labeling for NMR and luminescence-based studies [43], Gd.L^1^ has not been utilized as a protein label [46]. Therefore, we sought to compare its cysteine-labeling reactivity and performance with the widely used Gd.DO3A.

Gd.C12 contains the same reactive 4-nitropyridine arm as Gd.L^1^; however, the three chiral amide arms produce a spin label with an overall 3+ charge [44]. The chiral amide arms of Gd.C12 are important for use in NMR experiments as a paramagnetic tag, as this produces a single stereoisomer and prevents peak doubling in NMR spectra. The suitability of Gd.C12 for DEER distance measurements has been demonstrated previously [44]; however, we wished to compare this bulkier label with the smaller spin labels Gd.L^1^ and Gd.DO3A. In earlier work, the results from DEER-derived distance distribution comparisons of Gd.DO3A and Gd.DO3MA, where the latter label has methyl groups on the acetate arms, showed minimal differences [25]. However, we were curious as to whether the even greater size of Gd.C12 would lead to changes in the distance distributions obtained and whether the overall 3+ charge on the label would lead to any noticeable alterations in labeling efficiency.

The Materials and Methods section of the paper provides a summary of the sample preparation and the parameters used for the three spectrometers. The Results and Discussion section of this paper provides the outcomes and observations on the comparative sensitivity of the instruments, the robustness of Gd(III)–Gd(III) DEER measurements across the three labels and three spectrometers, and comments on labeling efficiency of the labels for CaMM13 34C 146C.

## Materials and Methods

### Spin Labels

MTSL was synthesized as previously reported and was a kind gift from Professor Dr Tamás Kálai at the University of Pécs, Hungary [47]. Gd.DO3A was synthesized as previously reported [25]. Gd.C12 was synthesized as previously reported [44]. The synthesis of Gd.L^1^ is presented in the Supporting Information, the L^1^ ligand was previously reported [43, 46], however the synthetic route has been modified.

### Calmodulin-M13 (CaMM13) 34C 146C Construct Design, Expression and Purification

The construct and expression protocol are provided in the Supporting Information.

### CaMM13 34C 146C Spin Labeling

MTSL: 80 µM freshly purified CaMM13 was incubated overnight at room temperature with five times excess of MTSL (from 50 mM DMSO stock) to protein in 500 µL buffer A (40 mM HEPES and 150 mM NaCl, pH 7.5). The sample was concentrated and cleaned using an Amicon Ultra 0.5 mL, 10 kDa membrane, centrifuge concentrator.

Gd(III) spin labels: The Gd(III) spin label stocks were made in buffer A with a concentration of 100 mM. Freshly purified CaMM13 was incubated overnight at room temperature with ten times excess of Gd(III) spin label to protein in buffer A. For Gd.C12 and Gd.L^1^ labeling, the concentration of protein was 2.3 mM. Gd.DO3A labeling was carried out with 1.4 mM protein (HiPER) and 2.6 mM protein (Q-band). The third Gd.DO3A-labeled protein labeled was at WIS with 1.4 mM CaMM13 that had been transported on dry ice with 50% v/v glycerol (the glycerol was removed prior to the labeling procedure). Following the labeling reactions, the samples were diluted to 400 µM in a final volume of 100 µL ready for the biotin-streptavidin purification protocol. At this stage, the product was checked with ESI–MS (Figure S2).

Biotin-streptavidin clean-up [48]: To remove unlabeled and single-labeled protein, the spin-labeled samples (with the exception of the Gd.DO3A-labeled protein measured at WIS) were incubated for 2 h at room temperature on a tabletop rotator with a 1:1 molar concentration (400 µM) of a biotin-maleimide (Sigma Aldrich) from a stock solution of 15 mM in DMSO, which binds to the free cysteines. After the incubation, the samples were made up to 1 mL in buffer A and loaded on a 1 mL HiTrap Streptavidin HP column. The protein was eluted in one mL of buffer A and concentrated down to 100 µL. The product of the clean-up was checked by mass spectrometry (Figure S2).

D_2_O solvent exchange and storage: The labeled protein was buffer exchanged into a D_2_O solution of 40 mM HEPES, 150 mM NaCl, pH 7.5 buffer and approximately 400 µM protein using a desalting Micro Bio-Spin™ 6 Column. This buffer was used for any dilution steps in the final sample preparations. With the exception of the Gd.DO3A-labeled protein measured at the WIS where the protein concentration was assumed correct, the protein concentration was determined through Bradford assay. The spin-labeled protein was stored in small aliquots at − 20 °C.

Q-band DEER samples: The MTSL-labeled protein was diluted to 40 µM in 60 µL containing 50% glycerol-d_8_ and 5 mM CaCl_2_. For the Gd(III) spin-labeled protein, the samples were made up to 20 µM in 60 µL containing 50% glycerol-d_8_ (v/v) and 5 mM CaCl_2_. The total volume was loaded into the quartz EPR tube.

WIS DEER samples: The sample was diluted to give 80 µM protein in 12 µL containing 30% glycerol-d_8_ (v/v) and 5 mM CaCl_2_. 3 µL was loaded into the quartz EPR tube.

HiPER DEER samples: The sample was 20 µM protein in 90 µL containing 50% glycerol-d_8_ (v/v) and 5 mM CaCl_2_. The total volume was used in the EPR experiment in a fluorinated ethylene propylene (FEP) tube.

### EPR Experiments

EPR experiments were carried out at W-band with the spectrometers described earlier and in the Supporting Information. Experiments were usually carried out at 10 K with a small additional study on the HiPER spectrometer at 6 K. Q-band EPR experiments were carried out with the high-power (150 W) Q-band (34 GHz) Bruker Elexsys E580 with an ER 5106QT-2w cylindrical resonator. The echo-detected EPR spectra were obtained using the pulse sequence (Hahn-Echo): π/2-τ-π-τ-echo. The echo decay spectra were acquired using the Hahn-Echo as in the experiment above, yet here we measure the echo intensity at the maximum (CT, the only case for the WIS and Q-band measurements) or away from the maximum (OffCT) of the Gd(III) signal as a function of τ. DEER traces were measured using the dead-time free four-pulse DEER sequence: π/2_νobs_ – τ_1_ – π_νobs_ – (τ_1_ + *t*) – π_νpump_ – (τ_2_ − *t*) – π_νobs_ – τ_2_ – echo (Fig. 1A) [6]. The Supporting Information contains further details of the measurement parameters and tables of the variables (Tables S1 to S5, and Figure S3).

### Analysis of EPR Data

The sharp central transition of the echo-detected field-swept spectra was analyzed to give an estimate of the FWHH by interpolating the collected data in Matlab. Decay curves were normalized to their maximum and the 10% remaining echo point was used as a measure of the echo decay time. The DEER traces were analyzed using Tikhonov regularization in DeerAnalysis2022 (MatLab2023a) [49], removing around 800 ns of data at the end of the trace, and fitting the background assuming a homogenous distribution. The Tikhonov regularization parameter was determined by the software. The variables are presented in Tables S1 to S5. To provide a measure of distance distribution uncertainty “Validation” was carried out by adding 50% white noise and varying the start and end points of the background fits using the default variables.

### Predicting the Spin Label Distribution

Computational simulations of the distance distributions were made with the python-based package chiLife on the 2BBM.pdb structure [42, 50, 51]. The rotamer libraries for Gd.DO3A and MTSL tags with off-rotamer sampling (5000 samples) were used [50].

## Results and Discussion

### General Remarks on Sample Preparation

CaMM13 was successfully expressed and spin labeled with three Gd(III) spin labels (Gd.DO3A, Gd.L^1^, Gd.C12) and MTSL. All three of the Gd(III) spin-labeled samples had their double-spin labeled protein purity improved by labeling the remaining free cysteines with biotin-maleimide and removing these from the solution using a streptavidin-containing column (Figure S2) [48]. The samples employed for the HiPER and Q-band measurements were in 50:50 (v/v) D_2_O buffer:glycerol-d_8_, whereas those measured on the WIS spectrometer were in 70:30 (v/v) D_2_O buffer:glycerol-d_8_.

### Echo-Detected EPR Spectra and Echo Decays

**Fig. 2:**
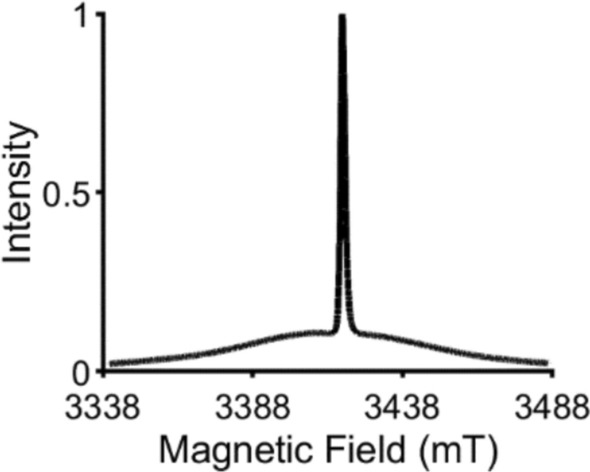
Normalized intensity echo-detected field-swept (ED-FS) spectrum for Gd.C12-spin-labeled CaMM13 34C 146C acquired at 95 GHz

An example W-band echo-detected field swept (ED-FS) spectrum of Gd.C12-labeled CaMM13 in the presence of Ca(II) is shown in Fig. 2. This shows the expected broad spectrum of the Gd(III) with the narrow central transition. Figure S4 shows the ED-FS spectra of the other Gd(III) spin-labeled CaMM13 measured at Q- and W-bands. The full-width at half-height (FWHH) of the central transition was determined and presented for the three spectrometers in Table 1. The FWHH of the Gd(III) spin-labeled protein for the two W-band spectrometers follows the trend Gd.DO3A >> Gd.L^1^ > Gd.C12. This trend reflects differences in the ZFS of the protein-bound tags. There are disparities between the HiPER and WIS FWHH values, which may be due to differences in the samples and measurement methods. The FWHH trend that the result for Gd.DO3A is greater than Gd.L^1^ is reflected in the Q-band measurement, though the ratio is not the difference between W- and Q-band frequencies: the Q-band ED-FS spectra appear more narrow than a multiplication of the W-band results by the W-:Q-band spectrometer frequency ratio predicts, as seen before [18].

**Table 1:** FWHH (full-width at half-height) for the central transition of the ED-FS spectra and the *T*_10%_ (time for 10% remaining echo in the echo decay experiment) measurements from the three spectrometers for CaMM13 labeled with Gd.C12, Gd.L^1^ and Gd.DO3A. Field-domain spectra with the FWHH marked are presented in Figure S4 and echo-decay curves are presented in Figure S5. “WIS” indicates the spectrometer at the Weizmann Institute of Science and “HiPER” is the W-band spectrometer at the University of St Andrews. CT is shorthand for measurements with the echo-detected decay pulse sequence set to the maximum of the central transition; OffCT is shorthand for measurements taken 300 MHz higher frequency than the maximum of the central transition peak

		FWHH/mT	Measured at	*T*_10%_/µs
WIS	Gd.C12 Gd.L^1^ Gd.DO3A	1.8 2.4 3.1	CT CT CT	11.6 14.0 14.2
HiPER	Gd.C12	1.6	CT OffCT	12.9 13.7
	Gd.L^1^	1.9	CT OffCT	16.1 15.7
	Gd.DO3A	4.0	CT OffCT	20.7 19.3
Q-band	Gd.L^1^ Gd.DO3A	4.8 6.2	CT CT	16.2 15.4

As the phase memory time (*T*_m_) is an important factor in the sensitivity of DEER, we also measured the echo decay for all spin labels, and these are presented in Figure S5. Table 1 presents the time for the 10% remaining signal (*T*_10%_) in the echo-decay measurements for the Gd(III) spin-labeled CaMM13 samples. The *T*_10%_ for the different spin labels measured at W-band indicate, in an albeit small sample set, that the Gd.C12 may have a shorter phase memory time than Gd. L^1^ or Gd.DO3A. The* T*_10%_ values for the WIS samples are consistently shorter because of the higher concentrations (80 µM compared to 20 µM for the other spectrometers) and the lower amount of glycerol. The glycerol is present to enable a good glass to be formed upon freezing, this is generally easier for smaller volume samples or for higher volumes of glycerol. A lower quality glass upon freezing may result in islands of higher local concentrations, i.e. increased spin–spin interactions. For HiPER it is essential to have a very good glass, so 50% glycerol was used, compared to 30% v/v for the WIS samples; 50% glycerol is also used in the Q-band measurements presented here.

The HiPER results for *T*_10%_ away from the central transition (OffCT) are similar to those taken on the central transition. For Gd.C12-labeled protein, the OffCT result is longer than the CT result. These results for Gd.C12 are contrary to the expectation that the decay rates measured away from the ${\text{m}}_{\text{s}} = - 1/2$ to ${\text{m}}_{\text{s}} = + 1/2$ transition are faster [52]. Further work would be required to determine the origin of this effect, but its existence here means that the OffCT DEER SNR compared to the OnCT DEER is not greatly affected by differences in decay rate.

### DEER Measurements

#### Distance Distributions and Modulation Depth

**Fig. 3:**
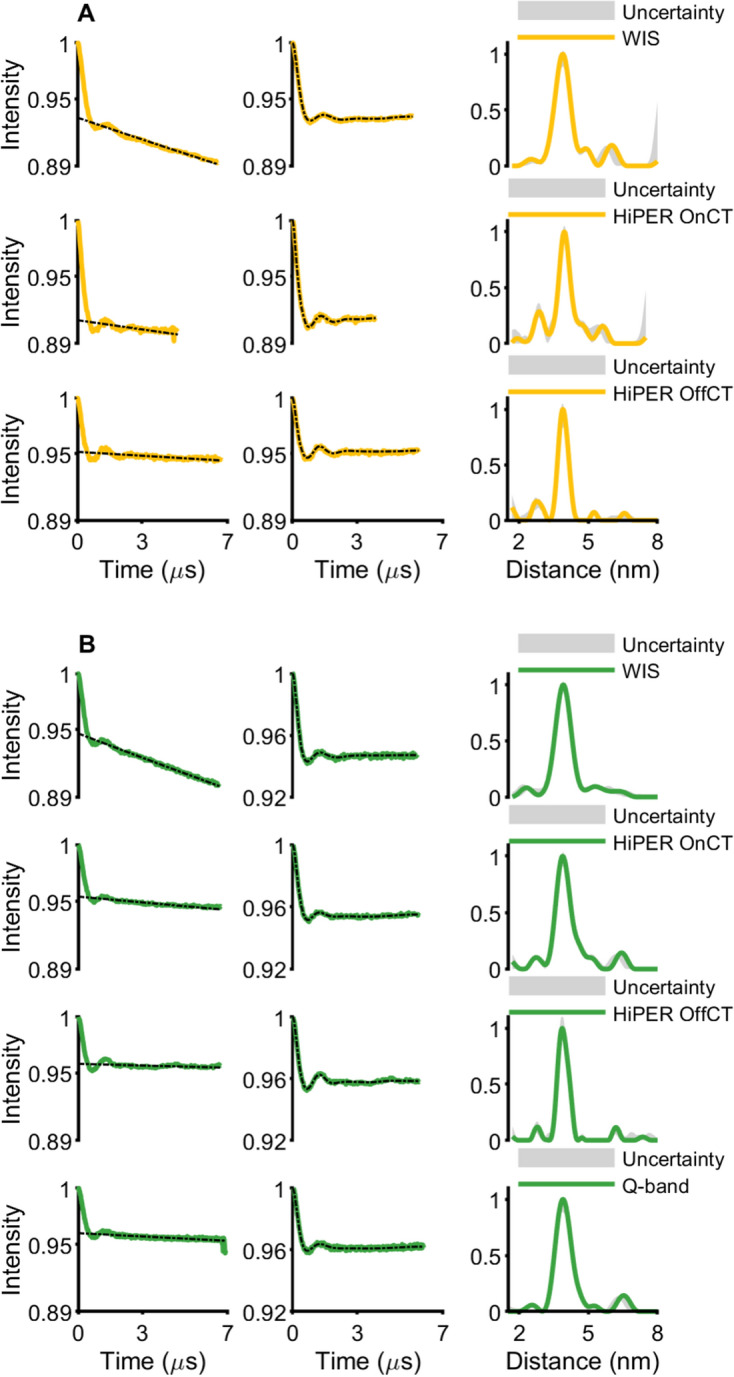

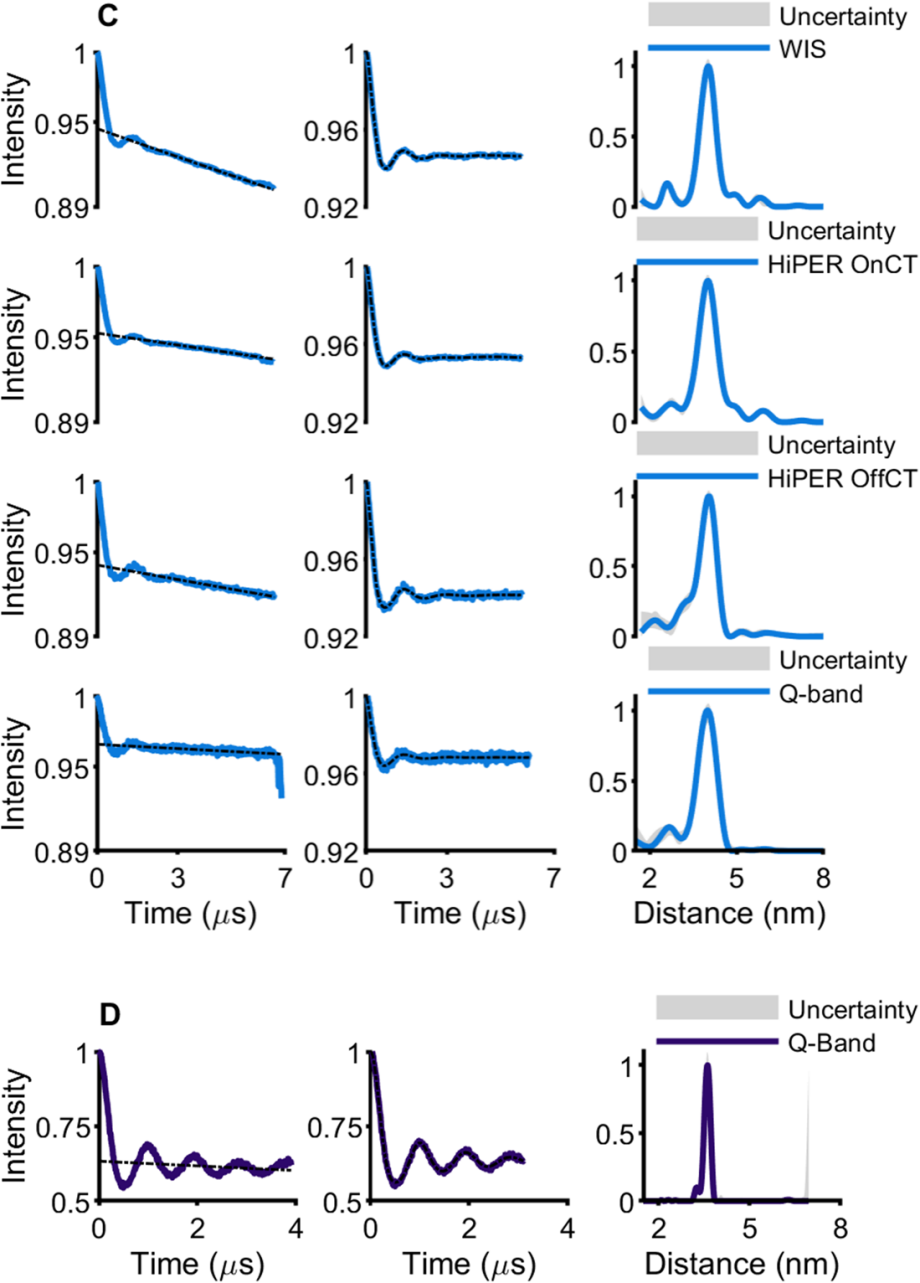
Experimental results from DEER for spin-labeled CaMM13 34C 146C showing the time traces, background corrected result from DeerAnalysis and the associated distance distribution. **A** Gd.C12 measured at W-band; **B** Gd.L^1^ measured at W- and Q-band, **C** Gd.DO3A measured at W- and Q-band, **D** MTSL measured at Q-band

**Fig. 4:**
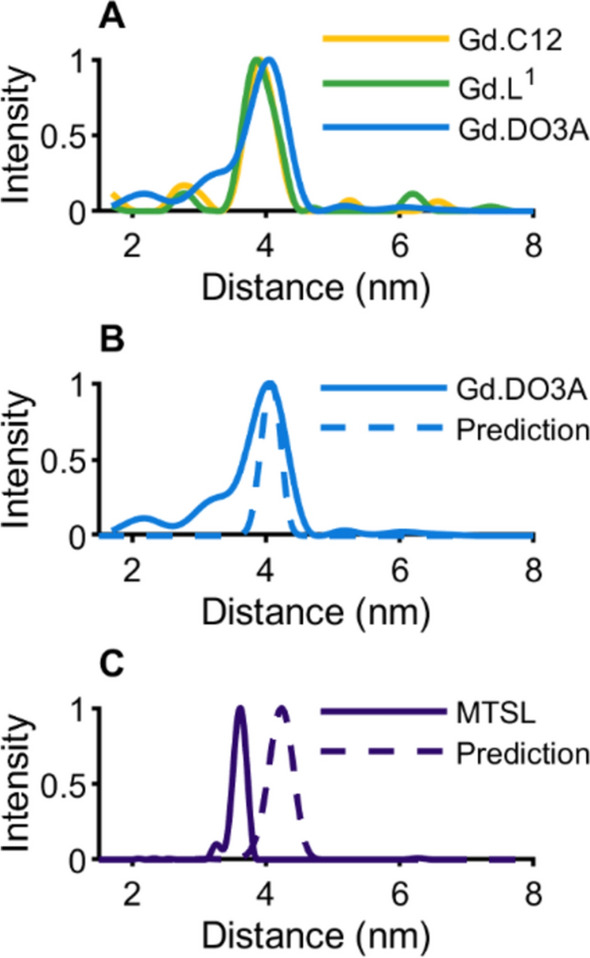
Comparison of DEER-derived distance distributions for the spin-labeled CaMM13 and in-silico modeling prediction from chiLife. **A** Overlay of HiPER OffCT results. **B** HiPER OffCT Gd.DO3A result compared to the model. **C** Q-band MTSL result compared to the model

Four-pulse DEER measurements were performed on all the spin-labeled CaMM13 34C 146C samples. The temperature was 10 K, which was previously shown to be optimal for W-band based on the intensity of the CT and the phase memory time [2]. The Q-band measurements were also taken at this temperature. Wideband excitation away from the central transition may mean that a lower measurement temperature may be optimal at W-band, and so to check this hypothesis measurements with Gd.DO3A spin-labeled protein in HiPER were also measured at 6 K. The defining experimental parameters are given in Tables S1 to S4 (and Table S5 for HiPER with the Gd.DO3A-labeled protein at 10 K and 6 K). Figure S3 presents the positions of the pump and observer pulse bandwidths on the Gd(III) spectrum. Figure 3 shows the DEER time traces, the DeerAnalysis processed time traces, and corresponding distance distributions (for results from DeerAnalysis of HiPER data collected at 10 K and 6 K for Gd.DO3A-labeled protein see Figure S7). The most probable distances from the analysis of the DEER time traces are presented in Table 2. The distance distributions derived from the HiPER measurements taken with pump and observer pulses away from the central transition (“OffCT”) are shown for each of the Gd(III) spin labels and compared to calculations for predicted inter-spin label distances from chiLife using 2BBM.pdb in Fig. 4 [42, 50].

**Table 2:** The most probable distance in nm for the DEER-derived distance distributions presented in Fig. 3

	WIS/nm	HiPER/nm		Q-band/nm
		OnCT	OffCT	
Gd.C12	3.89	3.97	3.91	
Gd.L^1^	3.90	3.88	3.86	3.89
Gd.DO3A	4.02	4.00	4.04	4.00

The DEER time traces for all measurements exhibit good SNR, where modulations of the echo caused by the dipole–dipole coupling between the Gd(III) spin labels are clear (Fig. 3). The superimposed background on the DEER time trace is more pronounced for the measurements at the WIS and this is attributed to the higher concentration of the sample and the lower glycerol concentration [53]. However, the derived distance distributions seem to be unaffected by this effect.

The obtained distance distributions (Fig. 3) are comparable for all Gd(III) measurements and to highlight the differences between the distance distributions we present (in Figure S6) quartile-quartile (Q–Q) plots. The distance distributions from the WIS and from the OnCT HiPER measurements are expected to be almost the same since the positions of the observe and pump pulses are similar. This is borne out for the Q–Q plots for Gd.L^1^ and Gd.DO3A. For Gd.C12 the HiPER distance distribution is narrower, but it does show a potential ghost peak at a shorter distance, which could be a consequence of the calculated Tikhonov regularisation parameter being smaller in this case (see Table S1). The Q-band data (i.e. Gd.L^1^ and Gd.DO3A) have similar distance distributions to the WIS and HiPER OnCT results.

The three Gd(III) labels give similar modal distances (Table 2, Fig. 4A, Figure S6), coinciding at approximately 3.9–4 nm. The results indicate a slightly different modal distance for the Gd.DO3A-labeled samples. The differences between the different spectrometers are within experimental error. The results agree very well with the chiLife modeling for Gd.DO3A on 2BBM.pdb (Fig. 4B). The MTSL distance distribution most probable distance is shorter than the Gd(III) spin label results. Notably the MTSL-MTSL distance distribution is very narrow for the CaMM13 34C 146C (Fig. 4C). The change of distance and distribution of the experimental result compared to the corresponding chiLife prediction (also Fig. 4C) and the Gd(III) spin label results is likely to be an effect of the very restricted conformations for MTSL on the protein, perhaps due to specific interactions with neighboring amino acids, not predicted by chiLife. The Gd(III) labels do not appear to inhabit such a restricted landscape and hence have broader distributions and a slightly longer most-probable distance.

In general, we do not see systematic differences in the modulation depth between the two W-band spectrometers with OnCT pumping (Table 3). The modulation depth is usually the highest for Gd.12, consistent with its more narrow central transition which allows for more spins to be excited by the pump pulse. The modulation depth was reduced in the Q-band DEER measurements, which was as expected due to bandwidth limitations. Nevertheless, the Q-band measurements using standard rectangular pulses and pumping on the central transition produced a DEER time trace which was adequate for distance analysis at the 4 nm range and gave comparable distance distribution results to the W-band measurements after sufficient averaging.

**Table 3:** The modulation depth (given by DeerAnalysis2022), SNR calculated for a single scan per mole (absolute sensitivity) and SNR for a single scan per concentration (concentration sensitivity) for the DEER measurements

			Modulation depth	Absolute sensitivity/nmol^−1^	Concentration sensitivity/mM^−1^
WIS	Gd.C12 Gd.L^1^ Gd.DO3A		6.8% 5.3% 5.4%	0.55 0.54 1.4	1.7 1.6 4.3
HiPER	Gd.C12	OnCT OffCT	8.9% 4.9%	0.20 0.16	18 15
	Gd.L^1^	OnCT OffCT	4.6% 4.2%	0.39 0.45	36 40
	Gd.DO3A	OnCT OffCT	4.7% 5.9%	0.12 0.12	10 11
Q-Band	Gd.L^1^ Gd.DO3A		4.0% 3.4%	0.26 0.11	16 6.3

The values are per protein concentration where each protein was doubly spin labeled

The measurements taken away from the central transition (OffCT, HiPER) have slightly more pronounced modulations, which often manifests as a narrower distribution of the most prominent distance peak for Gd.C12 and Gd.L^1^. This is consistent with there being some contribution from pseudo-secular terms in the dipolar coupling at this 4 nm distance, which can be neglected if the measurement does not use the central transition [13]. The results for Gd.DO3A are more consistent across the measurements, and this may be because of the larger FWHH CT (and therefore larger ZFS) of this label than for Gd.C12 and Gd.L^1^. This reduces the contribution from the pseudo-secular terms. These observations are confirmed by the Q–Q plots in Figure S6.

Surprisingly, the OffCT data from HiPER do not show a markedly different modulation depth compared to the OnCT data. For Gd.DO3A the modulation depth is even slightly increased. The HiPER pulses were modified for each experiment to give good modulation depth and represent what is possible: The parameter space was not sufficiently explored to be certain that individual pulse schemes were fully optimized and pulse schemes were thus not necessarily consistent across each experiment. However, the observation remains that not measuring at the CT does not appear to be particularly detrimental for the modulation depth if chirped pump pulses are available.

#### Sensitivity Considerations

The DEER data show that the distance distributions obtained from three different spectrometers with similar pulse setups for the three different Gd(III) are highly similar, attesting to the robustness of Gd(III)–Gd(III) DEER. The main differences between the HiPER and WIS are expected to be in the signal-to-noise (SNR) aspects. The SNR is presented in Tables S1–S5 as the ratio of the modulation depth to the standard deviation of the noise. For the DEER measurements presented here, these values varied between 27 and 200 for the complete measurements.

**Fig. 5:**
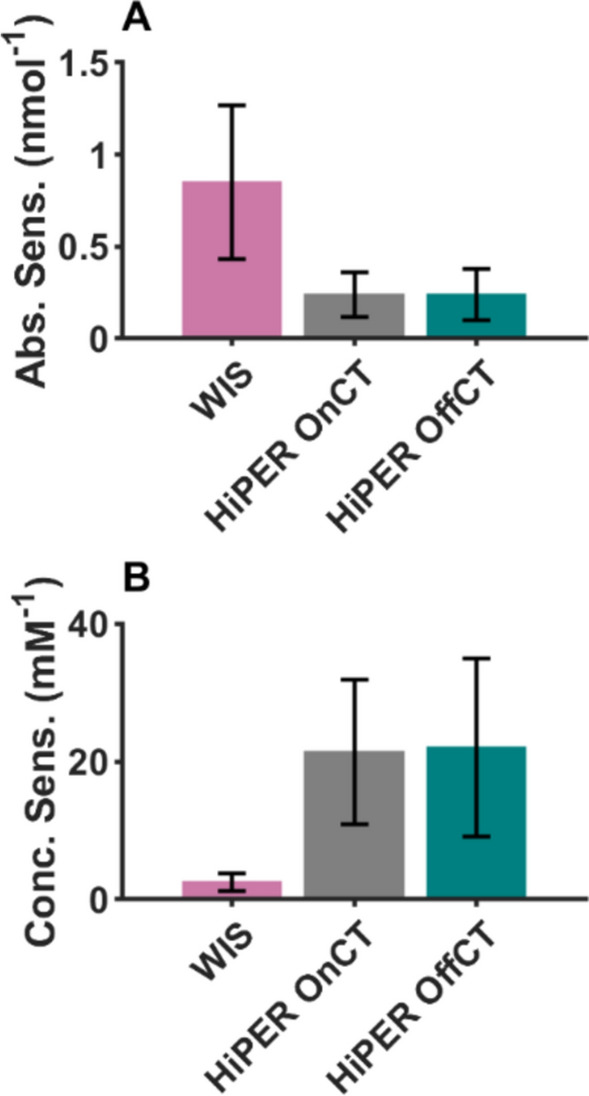
Bar graphs comparing the DEER SNR results showing averages and standard deviations for the three Gd(III) spin-labeled CaMM13. **A** SNR per mole (absolute sensitivity). **B** SNR per concentration (concentration sensitivity)

Tables 3, S1–5, and Fig. 5 present the SNR calculations for a single DEER time trace scan (which is calculated by dividing the SNR for the complete measurement by the square root of the total number of time traces). This highlights the differences in absolute and concentration sensitivity across the different spectrometer measurements. As expected from the spectrometer design, HiPER demonstrated greater concentration sensitivity than the instrument at the WIS, by about 8.5 times. This is principally due to the larger sample size. The instrument at the WIS had an absolute sensitivity around 3.5 times greater than HiPER.

The SNR for the OnCT and OffCT DEER measurements on HiPER are very similar and demonstrate that not only is the modulation depth recovered by using the shaped pulses on HiPER but also the observer pulses allow for the maintenance of SNR. One explanation is that we observed a smaller reduction in spin echo height in the presence of the OffCT pump pulse in this high spin system, an effect that still needs to be quantified and may warrant further investigation in future work [54, 55]. It is also partly due to the broad, flat frequency response of HiPER, and the use of Hamming-Sinc observer pulses, which give a small increase in echo height compared to rectangular or Gaussian pulses of comparable bandwidth.

The standard deviation of the SNR across the measurements was quite large. Notably, the Gd.DO3A-labeled CaMM13 measured at the WIS, and the Gd.L^1^ measured by HiPER had two to three times improved SNR over other samples. Conversely, the Gd.DO3A SNR results were the poorest for the HiPER and Q-band instruments. Interestingly, differences in relative SNR also correlated with the different observed linewidths for the same spin labels in the two laboratories (see Tables 1 and 3). It should be noted that the samples were prepared separately at each lab, but using the same protocol except for the final EPR sample composition and loading procedure (outlined below), so the observed differences in linewidth were unexpected and are not yet fully understood. Despite the shorter phase memory time of the Gd.C12-labeled protein, there was not an obvious difference in the SNR of the DEER measurements. This may be due to the counteracting balancing effect of the smaller ZFS for this label. The Q-band measurements have intermediate absolute and concentration sensitivity as compared to the results from the W-band instruments.

Measurements of Gd.DO3A-labeled protein at 6 K were performed in HiPER with the DEER results shown in Figure S7 and parameters in Table S5. Further work is required, but a significant enhancement in the SNR per individual scan, of order 2, was observed for both the OnCT and OffCT results, compared to measurements at 10 K.

#### User Experience

In terms of the user experience, the instrument at the WIS offers a particularly simple loading procedure and the very small volume inserted sample is measured soon after loading. The requirement for a good glass in HiPER has led to a protocol where sample loading is at 140 K, followed by several hours of cooling before measuring at 10 K (constrained by cooling power). This reduces the throughput of samples with respect to the Weizmann or Bruker Q-band spectrometer. However, the 3 mm OD (2 mm ID) plastic FEP tubes used in HiPER offer particularly easy sample handling when using viscous solutions, including we conjecture, cells, as long as the glass-forming qualities can be maintained. The Q-band spectrometer is also easy to use, though requires a longer measurement time for sufficient SNR.

### Observations Regarding the Spin Label Reactivity

Most of the spin-labeled protein samples were made just once. Therefore, it is not possible to draw definitive comparisons between spin label reactivity from our experimental results. However, in our hands with CaMM13 34C 146C, it appeared that Gd.DO3A may label more efficiently than Gd.C12 and Gd.L^1^, which was expected considering steric bulk and electronic reactivity factors [25, 26]. Further inspection of Figure S2 indicates that the charged Gd.C12 may label CaMM13 34C 146C at both cysteine sites with a higher yield than Gd.L^1^. However, in all cases, the biotin-streptavidin cleaning step was able to provide almost completely doubly-labeled protein. [48]

## Conclusions

We have presented a comprehensive and comparative study of four-pulse DEER at Q- and W-bands for three Gd(III) spin labels conjugated to cysteines in CaMM13 34C 146C. This represents the first demonstration of Gd.L^1^ as a spin label. The labels produced similar distance distributions with some broadening from pseudo-secular components of the dipolar interaction, which were removed when the central transition was not excited in the DEER experiment (HiPER OffCT). This work, therefore, highlights the robustness of DEER measurements using Gd(III) spin labels over different labels and different spectrometers. The measurement of each tag gave a comparable SNR with shorter decay times apparently being offset by smaller ZFS, and thus approximately equal numbers of detectable spins. W-band using the chirped and shaped pulses is superior to Q-band using rectangular pulses for efficiency of measurement. However, with patience, comparative distance distributions can be obtained at Q-band as well. Different spectrometer designs at W-band enable a focus on absolute or concentration sensitivity with the Weizmann Institute of Science spectrometer having superior absolute sensitivity over HiPER, and vice-versa for concentration sensitivity.

## Supplementary Information

Below is the link to the electronic supplementary material.

Supplementary file1 (PDF 2063 KB)

## Data Availability

The research data supporting this publication can be accessed at 10.17630/81ed7b0d-cb29-4851-9eee-bf4fb39e4f03.
