# Supplementary material for: Robustness and Sensitivity of Gd(III)–Gd(III) Double Electron–Electron Resonance (DEER) Measurements: Comparative Study of High-Frequency EPR Spectrometer Designs and Spin Label Variants
Source: Appl Magn Reson. 2025 Jan 3;56(5):591–611. doi: 10.1007/s00723-024-01741-0 (PMC12033185; doi:10.1007/s00723-024-01741-0)
Supplement: Supplementary file 1 — Supplementary file1 (PDF 2063 KB) [file 723_2024_1741_MOESM1_ESM.pdf]

Supporting Information

Robustness and sensitivity of Gd(III)-Gd(III) double electron-electron resonance (DEER) measurements: Comparative study of high-frequency EPR spectrometer designs and spin label variants.

Elena M. Mocanu[1], Yasmin Ben-Ishay[2], Lydia Topping[3], S. Ronan Fisher[1], Robert I. Hunter[1], Xun-Cheng Su[4], Stephen J. Butler[3], Graham M. Smith[1], Daniella Goldfarb[2], Janet E. Lovett[1]

1 SUPA School of Physics and Astronomy and BSRC, University of St Andrews, North Haugh, St Andrews, KY16 9SS, UK

2 Department of Chemical and Biological Physics, Weizmann Institute of Science, Rehovot 7610001, Israel

3 Department of Chemistry, Loughborough University, Epinal Way, Loughborough, LE11 3TU, UK

4 State Key Laboratory of Elemento-organic Chemistry, Tianjin Key Laboratory of Biosensing and Molecular Recognition, College of Chemistry, Nankai University, Tianjin, China

Contents:

Synthesis and characterisation of ligand L^1 and corresponding Gd(III) complex.
Further information on CaMM13 T34C T146C.
Further information for the HiPER spectrometer.
Further information and schematic for the Weizmann Institute of Science (WIS) W-band spectrometer.
CaMM13 mass spectrometry: methods and results.
Further information for the EPR and data analysis parameters.
Echo-detected field-swept (ED-FS) spectra.
Echo-detected decay curves.
Q-Q plots of the distance distributions.
CaMM13-Gd.DO3A HiPER results for 10 K and 6 K.
References.

Synthesis and characterisation of ligand L[1] and corresponding Gd(III) complex.

The synthesis of ligand **L[1]** was adapted from our previous work.[1]

2-(Hydroxymethyl)-4-nitropyridine (1)

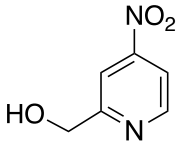

To a solution of commercially available 2-methyl-4-nitropyridine *N*-oxide (8.0 g, 52 mmol) in CH_2Cl_2 (130 mL) was added trifluoracetic anhydride (14.5 mL, 104 mmol) in CH_2Cl_2 (10 mL). The solution was stirred at room temperature for 72 hours, during which time the pale-yellow solution turned deep red in color. The solvent was removed under reduced pressure and resulting orange oil was dissolved in MeOH (80 mL) and saturated aqueous K_2CO_3 solution was added until the pH was approximately 8. The solution was stirred at room temperature for 24 hours. The solvent was removed under reduced pressure and the solid was partitioned between water (100 mL) and EtOAc (30 mL) and extracted with EtOAc (3 x 150 mL). The organic layers were combined, dried over magnesium sulfate, and concentrated under vacuum to give the crude material, which was purified by column chromatography (silica gel; 0–10% MeOH in CH_2Cl_2) to give the desired methyl alcohol **1** (4.30 g, 55%) as a pale-yellow solid. ^1H NMR (500 MHz, CD_3OD) δ: 8.77 (d, *J* = 5.4 Hz, 1H), 8.17 (s, 1H), 7.94 (d, *J* = 5.4 Hz, 1H), 4.78 (s, 2H), O-H signal not observed. ^{13}C NMR (125 MHz, CD_3OD) δ: 165.3, 154.2, 150.9, 114.6, 112.8, 63.9. The NMR spectral data are in agreement with those reported previously.[1] HRMS (ESI+) found 155.0451 $[M+H]^+$, $[C_6H_7N_2O_3]^+$ requires 155.0457. R_f = 0.48 (CH_2Cl_2/MeOH 96:4).

tert-Butyl protected ligand (2)

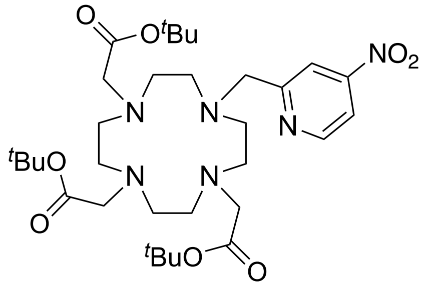

Step 1. To a solution of methyl alcohol **1** (0.60 g, 0.38 mmol) and DIPEA (99 µL, 0.57 mmol) in anhydrous $CHCl_2$ (4 mL) was added methansulfonyl chloride (28 µL, 0.36 mmol) and the resulting mixture was stirred at room temperature for 2 hours. The reaction mixture was washed with brine (10 mL), and the aqueous layer was extracted with CH_2Cl_2 (3 x 10 mL). The combined organic layers were dried over magnesium sulfate and concentrated under reduced pressure to give the mesylate ester (83 mg, 94%) as a yellow oil, which was used immediately in the next step.

Step 2: To a solution of *tert*-butyl protected DO3A (0.34 g, 0.65 mmol) in anhydrous CH_3CN (4 mL) was added potassium carbonate (260 mg, 1.9 mmol) and the mixture was stirred for 5 minutes. The mesylate ester of **1** (0.17 g, 0.72 mmol) in anhydrous CH_3CN (1 mL) was added and the mixture was stirred at 60°C for 24 hours. The reaction mixture was then cooled to room temperature and centrifuged for 3 minutes. The solution was decanted, and the solid pellets were washed twice with CH_3CN (2 x 5 mL). The combined organic layers were concentrated under reduced pressure and the crude material was purified by column chromatography (silica gel: 0-10% MeOH:CH_2Cl_2) to give the desired macrocyclic ligand **2** (266 mg, 63%) as a brown glass-like solid. ^{1}H NMR (400 MHz, $CDCl_3$) δ: 8.48 (d, *J* = 5.5 Hz, 1H), 7.91 (d, *J* = 1.7 Hz, 1H), 7.82 (dd, *J* = 5.5, 1.7 Hz, 1H), 3.56–2.04 (m, 24H), 1.38–1.22 (m, 27H). ^{13}C NMR (100 MHz, $CDCl_3$) δ: 173.0, 163.2, 154.3, 150.9, 115.9, 114.7, 82.0, 77.3, 59.2, 56.4, 55.6, 28.0. The NMR data are in agreement with those reported previously.[1] HRMS (ESI+) found 673.3891 $[M+Na]^+$, $[C_{32}H_{54}N_6O_8Na]^+$ requires 673.3901.

Ligand L¹

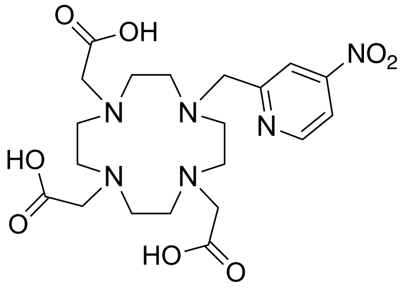

To a solution of protected ligand **2** (40 mg, 0.06 mmol) in CH_2Cl_2 (14.5 mL) was added trifluoracetic acid (1.6 mL, 0.02 mmol) and the mixture was stirred at room temperature for 72 hours. The mixture was concentrated under reduced pressure and the trifluoracetic acid was co-evaporated with CH_2Cl_2 to give the deprotected ligand **L¹** (28 mg, 98%) as a brown oil which was used without further purification. ^{1}H NMR (400

MHz, CD_3OD) δ: 8.87 (m, 1H), 8.21 (s, 1H), 8.09 (m, 1H), 4.41–2.89 (br m, 24H). HRMS (ESI+) found 483.2077 $[M+H]^+$, $[C_{20}H_{31}N_6O_8]^+$ requires 483.2203.

Complex Gd.L[1]

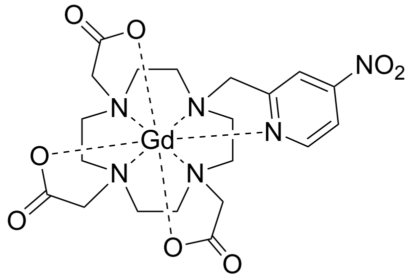

A solution of deprotected ligand **L[1]** (23 mg, 0.048 mmol) in H_2O (2.5 mL) was pH adjusted to 6.5 using 1M NaOH. $GdCl_3.6H_2O$ (1.05 equiv.) was added and the pH re-adjusted to 6.5 using 1M NaOH. The mixture was stirred at 60°C for 2 hours. Complete complexation was observed by LCMS analysis after this time. The water was removed by freeze drying and crude complex **Gd.L[1]** (30 mg, quant.) was obtained as a white solid. The complex was then purified by reverse-phase HPLC [XBridge C18 column, gradient: 0- 50% MeOH in 0.05% v/v formic acid, over 20 minutes at 4 mL per minute] to give **Gd.L[1]** (9 mg, 30%) as a white solid. Analytical RP-HPLC analysis [XBridge C18 column, 0% MeOH for 5 minutes followed by a gradient of 0–50% MeOH in 0.05% v/v formic acid over 10 minutes, at 1 mL per minute] revealed a single peak with a retention time of 12.85 minutes. HRMS (ESI+) found 638.1204 $[M+H]^+$, $[C_{20}H_{28}N_6O_8Gd]^+$ requires 638.1210.

Further information on CaMM13 T34C T146C.

Calmodulin-M13 (CaMM13) 34C 146C construct design, expression and purification.

The initial design for the human gene of Calmodulin bound through two glycine residues to the Rabbit MYLK2 calmodulin-binding domain (M13 peptide, CaMM13) was ordered from ThermoFisher and ligated into the pET14b vector (containing an N-terminal His-tag), and cloned into *E. coli* Top10 cells. The double mutant T34C and T146C was made through site-directed mutagenesis using the protocol for the NEB Q5 kit. For protein production, *E. coli* BL21(DE3) competent cells were transfected with the plasmid, and grown in 1 L of L-broth containing 50 μg/mL ampicillin and incubated overnight at 37°C in a shaker. The protein expression was induced at an OD600 of 0.8, by adding 1 mM IPTG and continued to incubate at 37°C for four hours. The bacteria was harvested by centrifugation at 6,000 RPM for 30 min and at 4°C. The pellet was resuspended in 30 mL lysis buffer (40 mM HEPES, 150 mM NaCl and a tablet of SIGMAFAST^{TM} Protease inhibitor Cocktail, pH 7.5), and lysed by using a cell sonicator. The product was isolated by centrifugation at 16,000 RPM for 30 min and 4°C, followed by filtration of the supernatant with a 0.45 μm pore size syringe filter. The supernatant was loaded on a 5mL Ni-NTA column equilibrated with buffer (40 mM HEPES and 150 mM NaCl, pH 7.5). The column was washed with the same buffer containing 20 mM imidazole to elute the protein bound nonspecific, while for the elution of the CaMM13 complex was used the buffer containing 500 mM imidazole. The fractions containing CaMM13 were concentrated down to 1 mL, using a Vivaspin20 concentrator with a 10 kDa membrane and treated overnight at 4°C with 50 mM DTT. The sample treated with DTT was loaded on a size exclusion column (HiLoad^{TM} 16/600 Superdex^{TM} 75 pg) equilibrated with 40 mM HEPES and 150 mM NaCl, pH 7.5 buffer. The same buffer in which the protein is eluted. The fractions containing the protein were collected and concentrated down to 1 mL. The protein concentration was determined by measuring the absorbance at 280 nm and applying the Beer-Lambert law for an extinction coefficient of 8605 $M^{-1}cm^{-1}$. The mass and integrity of the sample were checked on electrospray ionization mass spectrometry (ESI-MS).

Sequence for CaMM13 T34C T146C with His-tag at N-terminal end:

MGSSHHHHHHSSGLVPRGSHMADQLTEEQIAEFKEAFSLFDKDGDGTITTKELG**C**VMRSLGQNPTEAELQDMINEVDADGNGTIDFPEFLTMMARKMKDTDSEEEIREAFRVFDKDGNGYISAAELRHVMTNLGEKLTDEEVDEMIREADIDGDGQVNYEEFVQMM**C**AKGGKRRWKKNFIAVSAANRFKKISSSGAL

T34C primers: FWD: AGAACTGGGCtgcGTTATGCGTAG (5'-3'), REV: TTGGTGGTAAT GGTGC (3'-5'), annealing temperature: 61°C

T146C primers: FWD: TCAGATGATGtgcGCCAAAGGTG (5'-3'), REV: ACAAATTCTTCA TAGTTAACC (3'-5'), annealing temperature: 57°C

Further information for the HiPER spectrometer.

The spectrometer has been described before,[2] but has since been upgraded with a two channel 12 GSa/s M8190A AWG to provide up to 1 GHz instantaneous bandwidth. This AWG uses two coherent high-resolution NCOs (numerically controlled oscillators) to digitally synthesize coherent signals at 1.8 GHz in both signal and detection IF channels. In one channel digital IQ up-conversion, within the AWG, is then used to provide extremely high-fidelity signals over the range 1.3 GHz to 2.3 GHz. This signal is then finally upconverted to 93.5 to 94.5 GHz range in the spectrometer using a standard mixer operating with a high-fidelity fixed LO at 92.2 GHz, followed by SSB filtering. The LO at 92.2 GHz is generated using a low noise DRO at 7.6833 GHz followed by a 12-times frequency multiplier.

This scheme creates high fidelity signals at 94 GHz that can be both amplitude and phase modulated over a 1 GHz bandwidth. The signals are free of low-level artifacts often seen with commercial EPR systems that use lower frequency AWGs to generate IQ signals and use standard analogue IQ mixers. Shaped pulses can be used for both pump and probe pulses, with coherent detection.

The spectrometer is controlled via home-built software that offers considerable flexibility in specifying pulse sequences, pulse shapes, phase cycling and signal processing. In most experiments WURST ($n = 40$) chirp pulses were used as pump pulses and Hamming-Sinc pulses were used as detection pulses. The latter are highly selective, nominally single frequency amplitude modulated pulses that offer near rectangular excitation profiles in frequency space. Apart from high selectivity they also provide somewhat larger echo signals, compared to rectangular or Gaussian single frequency pulses for the same effective bandwidth. It is also possible to specify wideband chirped probe pulses, but these were not found to offer significant advantages relative to the Hamming Sinc pulses in the experiments described here.

For detection, EPR signals are first amplified using a 94 GHz LNA. They are then down-converted to an IF of between 1.3 GHz to 2.3 GHz, using the same 92.2 GHz LO used for up-conversion. A SSB filter is used to eliminate the amplified noise from the unwanted sideband centered at 90.4 GHz. The IF signal is then down converted to baseband using an analogue IQ mixer and a 1.8 GHz LO signal produced by the second channel of the AWG (which maintains coherence with the first channel).

Signals are then detected, averaged and signal processed using a fast digitizer in the standard manner. Both LOs use delay lines (at 1.8 GHz and 7.833 GHz) to equalize path differences between signal and detection channels.

It is also possible to coherently down-convert to an offset frequency (typically a few hundred MHz), followed by digitisation and signal processing. This has the significant advantage of being able to filter out dc signals and image frequencies originating from the IQ mixer down-conversion. This in turn helps to remove some of the deleterious effects of phase drift (due to thermal fluctuations in the cryostat) at 94 GHz. Further details will be given in a subsequent paper.

As samples are situated within a simple transmission line, measurements can be more sensitive to dielectric inhomogeneities in the sample compared to a standard resonator. It then becomes particularly important to form a good glass. FEP tubes are used with samples to avoid sample cracking at low temperatures often previously seen with quartz tubes, presumably due to differential thermal contraction. Samples are typically loaded (and removed) at a spectrometer temperature of 140 K and take several hours to stabilise at 10 K. Averaging times of up to 2 days have been successfully used in the past, although averaging times were shorter in the experiments described here.

Low loss, home-built, quasi-optical isolators are used to provide 90 dB isolation between source and sample holder and 60 dB isolation between detector and sample holder (all with negligible return loss). Induction mode operation typically provides more than 30 dB isolation (and up to 80 dB with careful adjustment at spot frequencies) between transmitter and detector. This significantly reduces the requirements for receiver protection and reduces deadtime. A fast ns laser switch can be used to eliminate dark noise from the high-power amplifier following the final pulse. Angled vacuum windows in the flow cryostat are used to virtually eliminate standing waves in the system and provide a flat frequency response even at kW power levels.

Further information and schematic for the Weizmann Institute of Science (WIS) W-band spectrometer.

The primary microwave (MW) sources of the MW channels are at 7.3±x GHz, which is multiplied by 13 to yield 94.9±x GHz. These are then fed into two independent MW channels with pulse-forming switches and phase shifters operating at the high frequency. One of the MW channels can also be controlled via an AWG as described earlier with some minor changes.[3] The AWG channel described in reference [3] has been modified (see Fig. S1) and instead of feeding the 7.3 GHz frequency into a x13 multiplier it is fed into a mixer with the output of an 87.6 GHz oscillator (Spacek PL6-876-5 phase-locked source, that consists of a DRO (dielectric resonance oscillator) followed by a 6x frequency multiplier, amplifier and filter). The output is fed into a high-pass filter, yielding the final frequency of 94.9 GHz.

Our setup currently can apply shaped pump pulses but not detection pulses. The two MW channels are combined and fed into the solid-state amplifier. The output of the amplifier is fed into a quasi-optical circulator (custom design by Thomas Keating), which is fed via a horn and a quasi-optical 90° turn into an oversized "lossless" corrugated waveguide, the output of which is fed again via a 180° quasi-optical into a horn which feeds into the cavity.[4] The cavity used is a cylindrical TE_{011} cavity made out of a brass tube plated with gold.[5] The cavity bandwidth is about 150 MHz, which gives a Q-value of ~630. The system is cooled via a closed cycle cryo-free system (Cold edge) with a minimal temperature of 6-7 K for DEER measurements. The system is stable allowing continuous data collection for up to 48 h. It allows rapid and easy sample change with about 1 hour cooling time back to 9-10 K.

The spectrometer is controlled by the SpecMan4EPR software which offers a simple user-friendly pulse programming language allowing for easy set-up of multi-dimensional experiments, CPMG detection.[6] Data are collected in transient mode, in which a full time-trace (up to 4 Mpoints) is saved with a maximal temporal resolution of 2 ns and echo integration is done post-measurement. The AWG card is controlled by a *LabView* program that has been written for this purpose and it receives its trigger from the spectrometer control software Specman.[3]

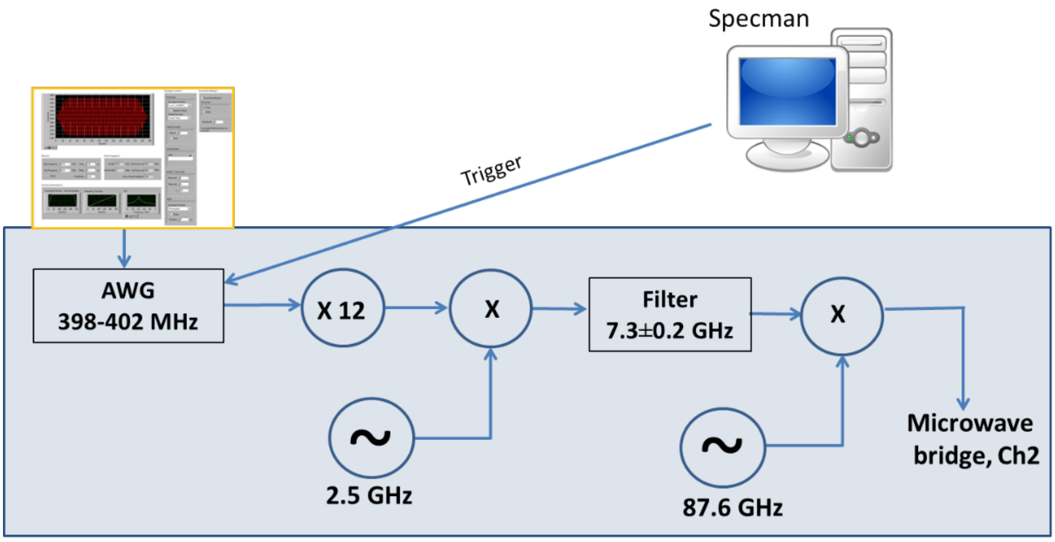

Figure S1 – Schematic of the AWG channel of the WIS spectrometer.

CaMM13 mass spectrometry: methods and results

Intact mass measurements were carried out at the University of St Andrews on a Waters Xevo G2TOF mass spectrometer with Waters Acquity LC. A volume of 30 µL of 1 µM sample was desalted online through a Waters MassPrep On-Line Desalting Cartridge, with a gradient elution of buffer A (95% water containing 1% formic acid:5% acetonitrile) to solvent B (5% water containing 1% formic acid:95% acetonitrile) with a flow rate of 0.2 mL/min and eluted directly into the MS. Data was acquired from 500-2500 m/z under positive ESI conditions. LeuEnk was used as an internal calibrant lock mass. The spectra across the elution peak were combined, and the charged ion envelope was deconvoluted using peak width at half height of the most intense charge state in the MaxEnt1 algorithm part of MassLynx V4.1 software. The results are shown in Figure S2.

The CaMM13-Gd.DO3A sample measured at WIS was spin labeled on site, and a local mass spectrometer was used to assess approximately full double labeling had occurred with no need for a biotin-streptavidin cleaning step (LC-MS results not shown).

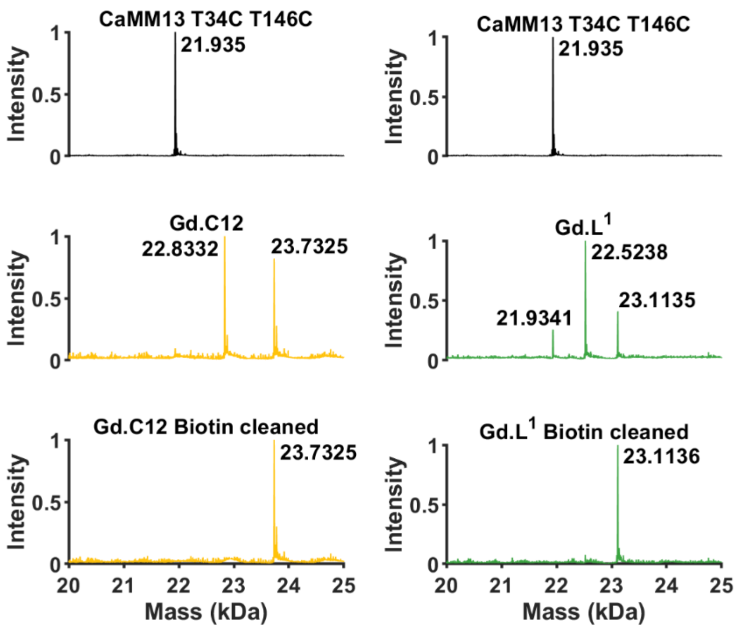

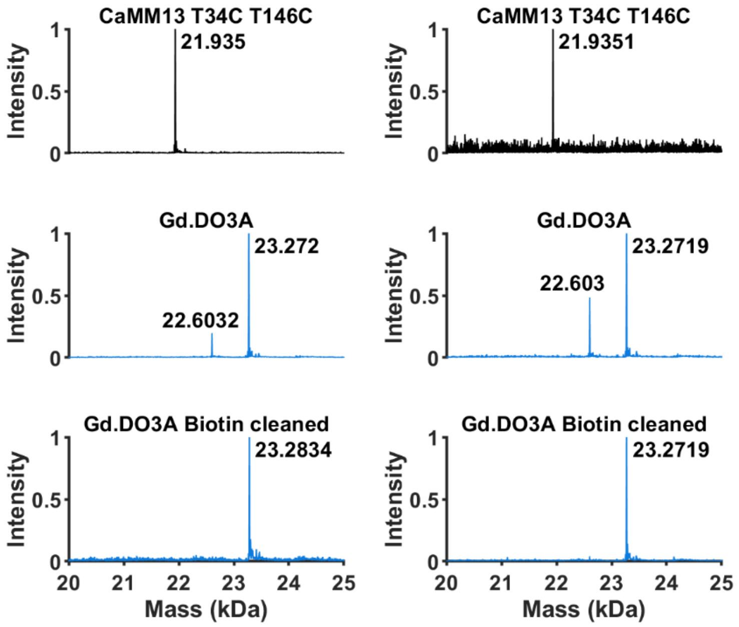

Figure S2 – LC-ESI mass spectra results for CaMM13 and spin-labeled CaMM13 before and after biotin-streptavidin cleaning. The masses are given in kDa. The results are for all the Gd.C12-labeled samples, all the Gd.L^1 – labeled samples, the first (LHS) Gd.DO3A result is for the samples measured at Q-band (the final sample was measured after buffer exchange into the DEER buffer, and the deuterium exchange accounts for the 11 Da additional mass) and the RHS result is for the sample measured on HiPER.

Further information for the EPR and data analysis parameters

ED-FS: The echo-detected field-swept (ED-FS) EPR spectra were obtained using the pulse sequence (Hahn-Echo): π/2-τ-π-τ-echo with a swept magnetic field and at 10 K. At WIS a two-step phase cycle (0,π) on the first π/2 pulse was used and the experimental parameters: $t_{\pi/2}$ =15 ns, t_{π} =30 ns, τ =500 ns (50 ns step), repetition time = 600 ns, and number of shots = 20. In HiPER, a similar Hahn echo sequence usually with a two-step phase cycle was used, with rectangular π/2 and π pulse lengths of either 6 ns and 12 ns, or 10 ns and 20 ns, 2000 shots per point and 500 μs shot repetition time. At Q-band the pulse lengths were $t_{\pi/2}$ =16 ns, t_{π} =32 ns, the τ =400 ns and shot repetition time was 1 ms (4 ms shot repetition time at 50 K for MTSL).

The echo decay curves were acquired using the Hahn-Echo and recording the echo intensity as a function of 2τ. Echo measurements were typically measured at the spectral maximum of the Gd(III) signal as a function of τ (94 GHz for HiPER) though data was also acquired on HiPER away from the central transition (OffCT at 94.3 GHz for the same field). At WIS, the experimental parameters were similar to the ED-FS EPR experiment with the exception of recording 50 shots, step time 50 ns and repetition time equal to 600 μs. In HiPER π/2 and π pulses of 8 and 16 ns were used with a 100 ns step size, 2000 shots per point and a shot repetition time of 500 μs. At Q-band the initial τ was set to 380 ns with a step of 50 ns (20 ns for MTSL at 50 K), the number of shots per point was sufficient for SNR, the shot repetition time was 10 ms.

Four-pulse DEER data were acquired with rectangular, chirped or hamming sinc truncation 4 (HS4) pulses with these listed along with the position of the observer and pump pulses and other experimental parameters in Tables S1 to S5. The pulse positions for the Gd(III)-labeled CaMM13 are shown on the corresponding ED-FS spectrum in Figure S3 (using frequency units). At WIS, *t* was incremented in steps of 20 ns ranging from -200 ns to the set evolution time. Four-step phase cycling was used. For HiPER the signals were typically down-converted to an offset frequency of 300 MHz, Fourier transformed and then digitally filtered in frequency space to remove dc offsets and image frequencies (close to matched filtering). The resultant signal is then inverse Fourier transformed and the absolute value recorded, without the need for phase cycling. This procedure removes any effects caused by slow phase drift of

the signal due to small temperature fluctuations in the flow cryostat. This procedure has been found to give consistent results over long averaging times, at the cost of a small reduction in SNR (from measuring the absolute value), and will be discussed in more detail in a subsequent publication. For all Q-band experiments *t* was incremented in steps of 8 ns, from 80 ns after the second observer pulse to 80 ns before the third observer pulse. The first delay, τ_1, was stepped from 400 ns to 496 ns in 24 ns increments to help remove any unwanted ^2H ESEEM (a procedure called tau-averaging in the parameter tables). A 16-step phase cycle was used.

The standard deviation of the DEER time trace SNR evaluation was calculated in Matlab by subtracting a smoothed signal from the raw trace. The smoothed signal was obtained by applying a moving mean filter and for consistency we used a window of 5 samples for all files. This method is similar by the one reported in literature.[4]

Table S1 – The experimental parameters for the three Gd.C12-labeled CaMM13 experiments.

Gd.C12	WIS	HiPER OnCT	HiPER OffCT
**Sample Properties**			
**Protein concentration c**	80 µM	20 µM	20 µM
**Volume of the sample V**	3 µL	90 µL	90 µL
**Glycerol**	30%	50%	50%
**DEER Parameters**			
**Temperature**	10 K	10 K	10 K
**No of shots**	25	2000	2000
**No of scans**	6829	20	20
**No of points**	290	250	350
**Phase cycling steps**	4	1	1
**Shot repetition time (SRT)**	200 µs	505.1 µs	502.9 µs
**Total measuring time**	21h17min	/	/
**Pump frequency**	94.975 GHz	93.9 GHz	93.6 GHz
	95.125 GHz	94.1 GHz	93.8 GHz
**Pump pulse shape**	Chirp	Chirp	Chirp
**Pump pulse total length (π)**	100 ns	50 ns	50 ns
**Observer frequency GHz**	94.85	94.3	94.3
**Observer pulse length (π/2)**	15 ns	8 ns	8 ns
**Observer pulse length (π)**	30 ns	16 ns	16 ns
**Tau1**	600 ns	400 ns	400 ns
**Tau2**	6 µs	5 µs	7 µs
**Tau averages**	0	0	0
**Data Analysis**			
**Modulation depth**	6.80%	8.90%	4.90%
**Window size for smoothed signal**	5	5	5
**Standard deviation**	0.00062142	0.0012112	0.0008282
**SNR of measurement (mod depth/Std deviation)**	109.4	73.5	59.2
**n (phase cycle*no shots*no scans)**	682900	40000	40000
**SNR (SNR of measurement/√n)**	0.13	0.37	0.30
**SNR per mol (SNR/(c*V)) nmol^{-1}**	0.55	0.20	0.16
**SNR per concentration (SNR/c) mM^{-1}**	1.7	18	15
**DeerAnalysis Parameters**			
**Maximum time (ns)**	5600	4680	6680
**Cutoff (ns)**	4800	3880	5880
**Zero time (ns)**	177	290	287
**Background (ns)**	1000	1100	1280
**Tikhonov regularization parameter**	126	40	79

Table S2 – The experimental parameters for the four Gd.L[1]-labeled CaMM13 experiments.

Gd.L[1]	WIS	HiPER OnCT	HiPER OffCT	Q-Band
		**Sample Properties**		
**Protein concentration c**	80 µM	20 µM	20 µM	20
**Volume of the sample V**	3 µL	90 µL	90 µL	60
**Glycerol**	30%	50%	50%	50%
		**DEER Parameters**		
**Temperature**	10 K	10 K	10 K	10 K
**No of shots**	500	1000	1000	30
**No of scans**	171	20	16	17
**No of points**	340	351	351	905
**Phase cycling steps**	4	1	1	16
**Shot repetition time (SRT)**	200 µs	502.3 µs	501.5 µs	1000 µs
**Total measuring time**	9h23min	/	/	/
**Pump pulse**	94.975 GHz	94.0 GHz	94.2 GHz Chirp	34.023 GHz
	95.125 GHz	100 MHz	94.5 GHz	/
**Pump pulse shape**	Chirp	HS4	Chirp	Rectangular
**Pump pulse total length (π)**	100 ns	60 ns	60 ns	12 ns
**Observer frequency GHz**	94.85	93.7	93.7	33.943 (υ 80 MHz)
**Observer pulse length (π/2)**	15 ns	HS4 60 ns	HS4 60 ns	13 ns
**Observer pulse length (π)**	30 ns	HS4 60 ns	HS4 60 ns	26 ns
**Tau1**	600 ns	400 ns	400 ns	400 ns
**Tau2**	7 µs	7 µs	7 µs	7 µs
**Tau averages**	0	0	0	5
		**Data Analysis**		
**Modulation depth**	5.30%	4.60%	4.20%	4.00%
**Window size for smoothed signal**	5	5	5	5
**Standard deviation**	0.00069671	0.00045767	0.00041287	0.00062334

**SNR of measurement (mod depth/Std deviation)**	76.1	100.5	101.7	64.2
**n (phase cycle*no shots*no scans)**	342000	20000	16000	/
**n_Q (Q-band phase cycle*no shots*no scans *tau averages)**	/	/	/	40800
**SNR (SNR of measurement/√n)**	0.13	0.71	0.80	0.32
**SNR per mol (SNR/(c*V)) nmol^{-1}**	0.54	0.39	0.45	0.26
**SNR per concentration (SNR/c) mM^{-1}**	1.6	36	40	16
**DeerAnalysis Parameters**				
**Maximum time (ns)**	6600	6680	6680	6888
**Cutoff (ns)**	5800	5880	5880	6088
**Zero time (ns)**	177	307	312	342
**Background (ns)**	960	1020	1320	1240
**Tikhonov regularization parameter**	200	126	63	2512

Table S3 – The experimental parameters for the four Gd.DO3A-labeled CaMM13 experiments.

Gd.DO3A	WIS	HiPER OnCT	HiPER OffCT	Q
		**Sample Properties**		
**Protein concentration c**	80 μM	20 μM	20 μM	20
**Volume of the sample V**	3 μL	90 μL	90 μL	60
**Glycerol**	30%	50%	50%	50%
		**DEER Parameters**		
**Temperature**	10 K	10 K	10 K	10 K
**No of shots**	25	5000	5000	30
**No of scans**	2549	100	20	20
**No of points**	340	351	351	905
**Phase cycling steps**	4	/	/	16
**Shot repetition time (SRT)**	200 μs	206.17 μs	207.9 μs	1000 μs
**Total measuring time**	9h19min	/	/	/
**Pump frequency**	94.975 GHz	94 GHz	94.2 GHz	33.993 GHz
	95.125 GHz	100 MHz	94.5 GHz	/
**Pump pulse shape**	Chirp	HS4	Chirp	Rectangular
**Pump pulse total length (π)**	100 ns	60 ns	100 ns	14 ns
**Observer frequency**	94.85 GHz	93.7 GHz	93.7 GHz	33.843 GHz (υ 150 MHz)
**Observer pulse length (π/2)**	15 ns	HS4 30 ns	HS4 30 ns	8 ns
**Observer pulse length (π)**	30 ns	HS4 60 ns	HS4 60 ns	16 ns
**Tau1**	600 ns	600 ns	600 ns	400 ns
**Tau2**	7 μs	7 μs	7 μs	7 μs
**Tau averages**	0	0	0	5
		**Data Analysis**		
**Modulation depth**	5.40%	4.70%	5.90%	3.40%

**Window size for smoothed signal**	5	5	5	5
**Standard deviation**	0.00030854	0.00031916	0.00084297	0.0012274
**SNR of measurement (mod depth/Std deviation)**	175.0	147.3	70.0	27.7
**n (phase cycle*no shots*no scans)**	254900	500000	100000	/
**n_Q (Q-band phase cycle*no shots*no scans *tau averages)**	/	/	/	48000
**SNR (SNR of measurement/√n)**	0.35	0.21	0.22	0.13
**SNR per mol (SNR/(c*V)) nmol^{-1}**	1.4	0.12	0.12	0.11
**SNR per concentration (SNR/c) mM^{-1}**	4.3	10	11	6.3
		**DeerAnalysis Parameters**		
**Maximum time (ns)**	6580	6580	6600	6872
**Cutoff (ns)**	5780	5780	5800	6072
**Zero time (ns)**	189	415	399	358
**Background (ns)**	920	1000	1440	840
**Tikhonov regularization parameter**	63	126	159	3162

Table S4 – The experimental parameters for the Q-band MTSL-CaMM13 measurement.

MTSL	Q-Band
**Sample Properties**	
**Protein concentration c**	20 µM
**Volume of the sample V**	60 µL
**Glycerol**	50%
**DEER Parameters**	
**Temperature**	50 K
**No of shots**	30
**No of scans**	2
**No of points**	530
**Phase cycling steps**	16
**Shot repetition time (SRT)**	4000 µs
**Total measuring time**	/
**Pump pulse**	34.024 GHz
**Pump pulse shape**	Rectangular
**Pump pulse length (π)**	14 ns
**Observer pulse**	33.944 GHz (υ 80 MHz)
**Observer pulse length (π/2)**	11 ns
**Observer pulse length (π)**	22 ns
**Tau1**	400 ns
**Tau2**	4 µs
**Tau averages**	5
**Data Analysis**	
**Modulation depth**	36.70%
**Window size for smoothed signal**	5
**Standard deviation**	0.0036329
**SNR of measurement (mod depth/Std deviation)**	101.0
**n (phase cycle*no shots*no scans)**	/
**n_Q (Q-band phase cycle*no shots*no scans *tau averages)**	4800
**SNR (SNR of measurement/√n)**	1.46
**SNR per mol (SNR/(c*V)) nmol^{-1}**	1.2
**SNR per concentration (SNR/c) mM^{-1}**	73
**DeerAnalysis Parameters**	
**Maximum time (ns)**	3896
**Cutoff (ns)**	3096
**Zero time (ns)**	330
**Background (ns)**	1024
**Tikhonov regularization parameter**	50

Echo-detected field-swept (ED-FS) spectra.

Gd.C12

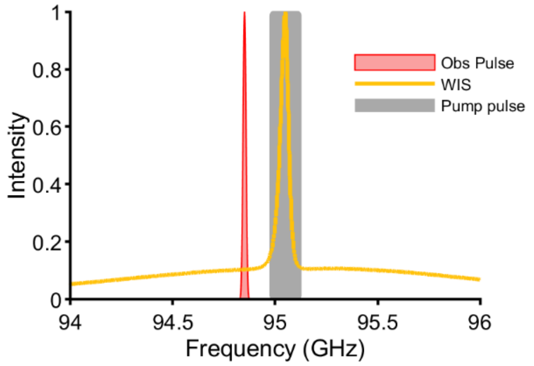

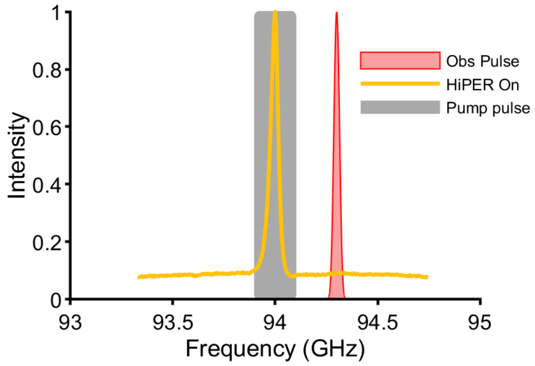

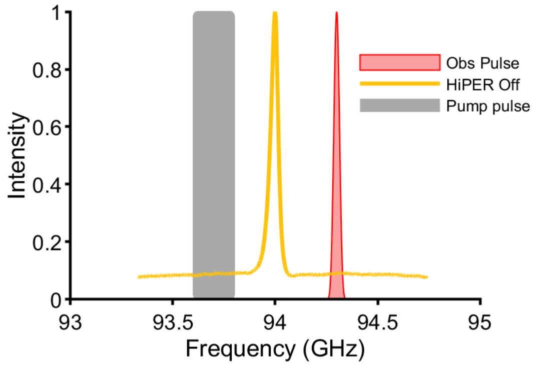

Gd.L^1

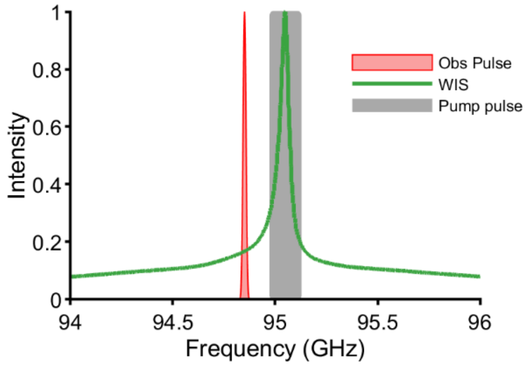

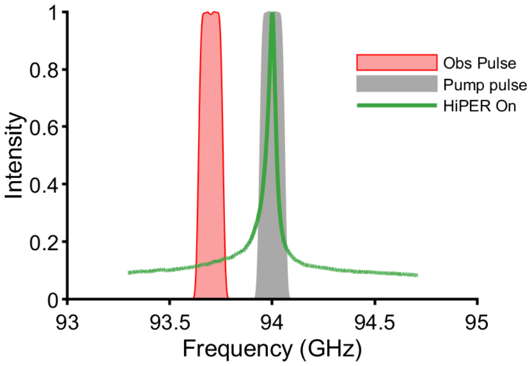

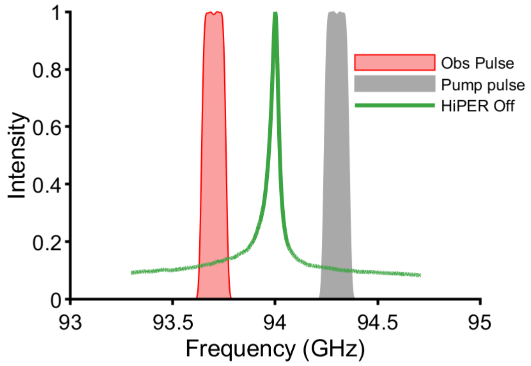

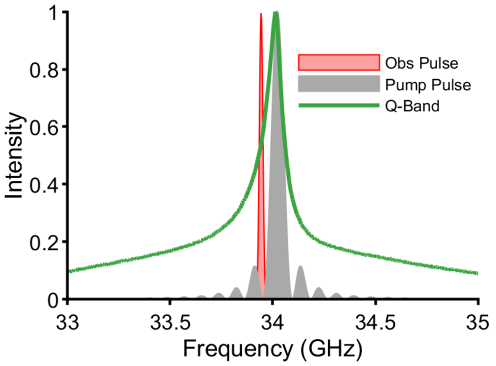

Gd.DO3A

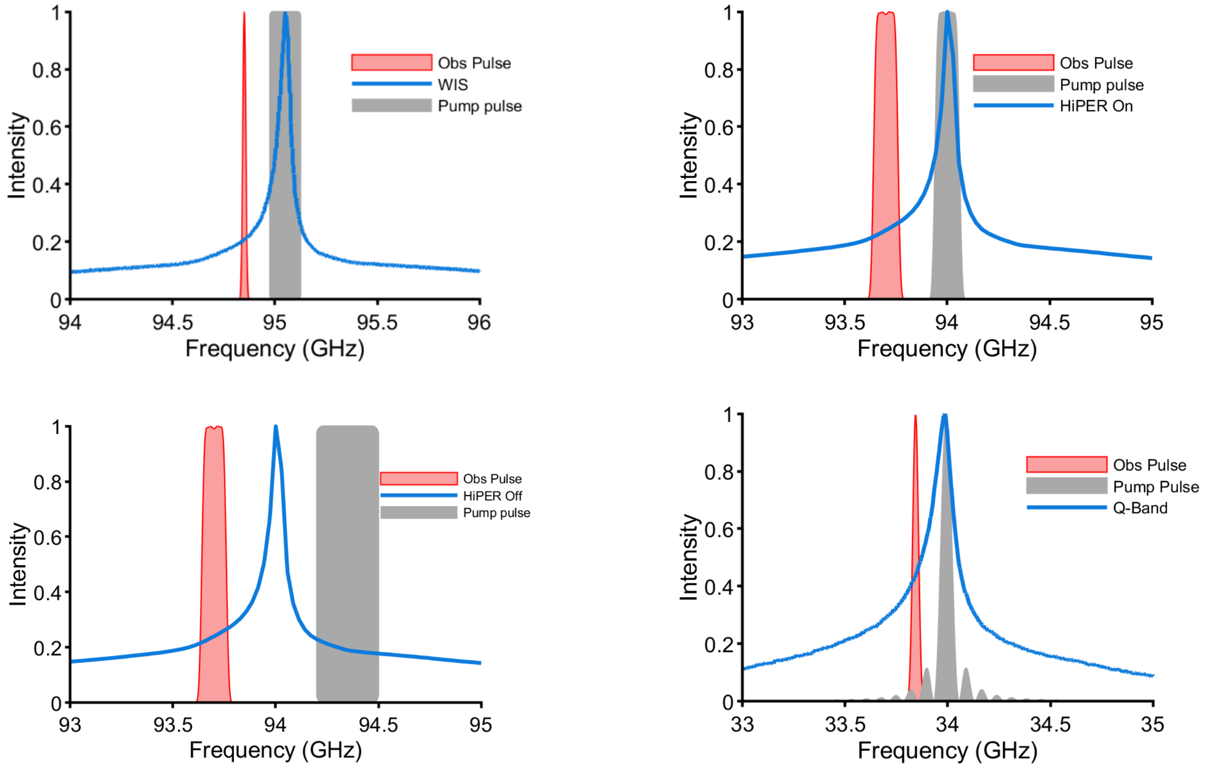

Figure S3 – ED-FS spectra of Gd(III) spin-labeled CaMM13. The x-axis is shown in frequency units and set to cover 2 GHz for each sub-figure. The bandwidth and positions of the experimentally used pump pulse and observer pulses and are represented in grey and red, respectively. In the case of an observer pulse sequence using rectangular pulses the bandwidth of the whole sequence was calculated and is shown.[7] Otherwise EasySpin was used to calculate the π pulse profiles.[8]

Gd.C12

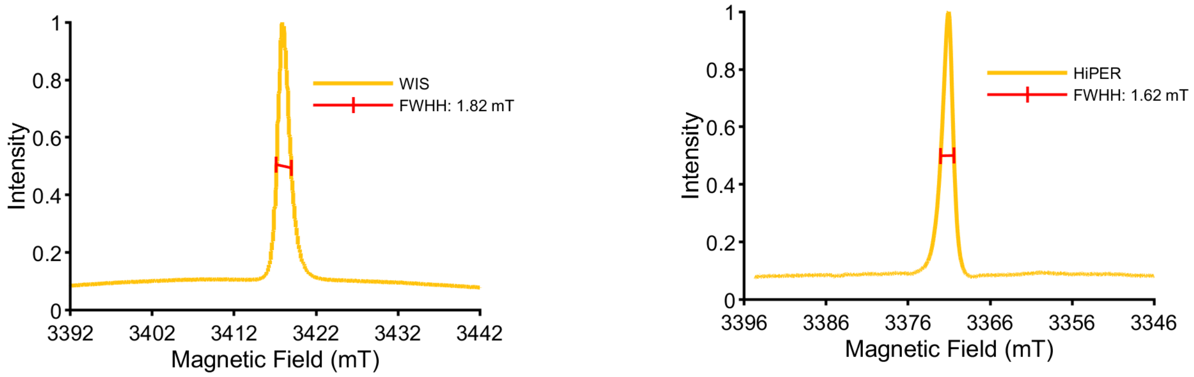
WIS
FWHH: 1.82 mT
Intensity
Magnetic Field (mT)
3392
3402
3412
3422
3432
3442
HiPER
FWHH: 1.62 mT
Intensity
Magnetic Field (mT)
3396
3386
3376
3366
3356
3346

Gd.L[1]

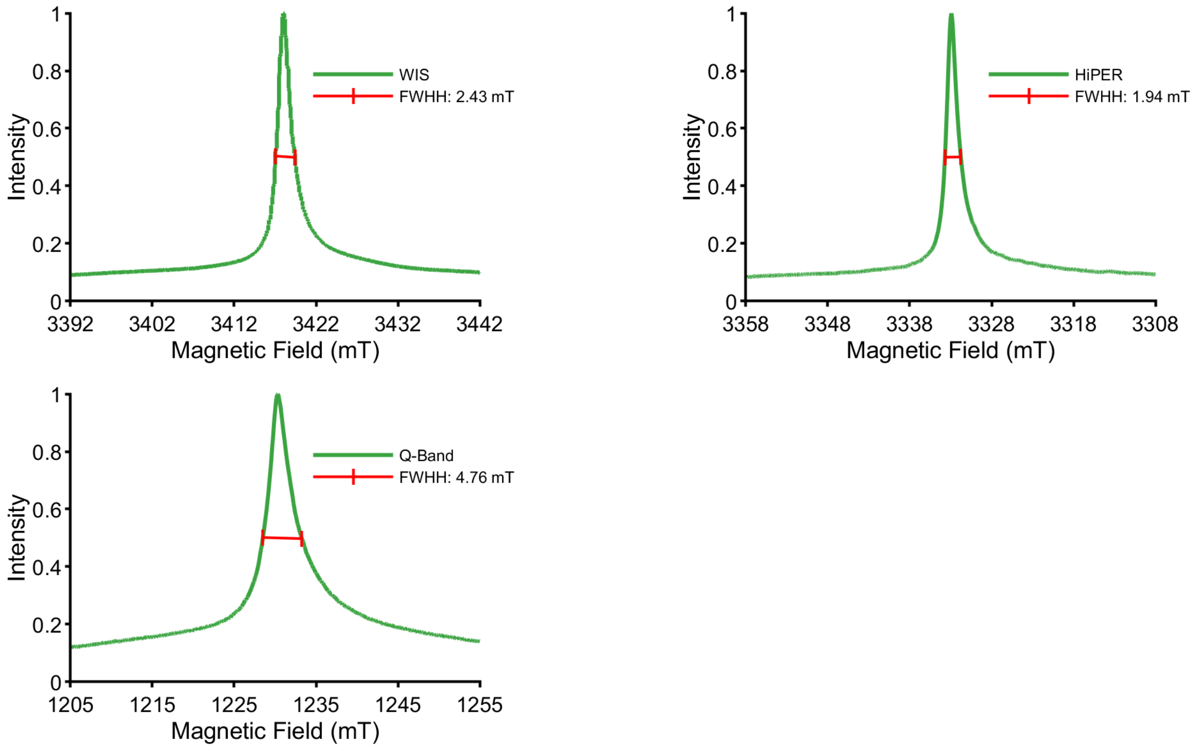
WIS
FWHH: 2.43 mT
Intensity
Magnetic Field (mT)
3392
3402
3412
3422
3432
3442
HiPER
FWHH: 1.94 mT
Intensity
Magnetic Field (mT)
3358
3348
3338
3328
3318
3308
Q-Band
FWHH: 4.76 mT
Intensity
Magnetic Field (mT)
1205
1215
1225
1235
1245
1255

Gd.DO3A

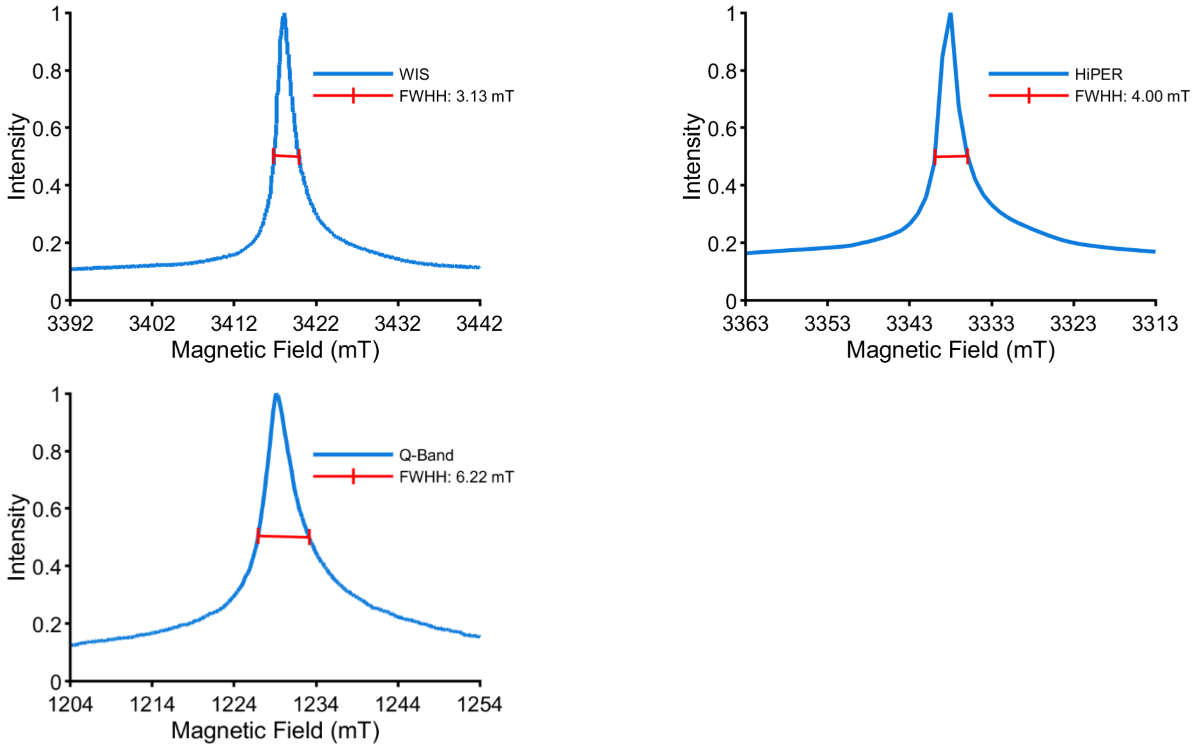

Figure S4 – ED-FS spectra Gd(III) of spin-labeled CaMM13. The full-width at half-height (FWHH) of the central transition is calculated in mT and represented with a red horizontal line. To calculate the FWHH, all data were interpolated to 9000 points and normalized.

Echo-detected decay curves.

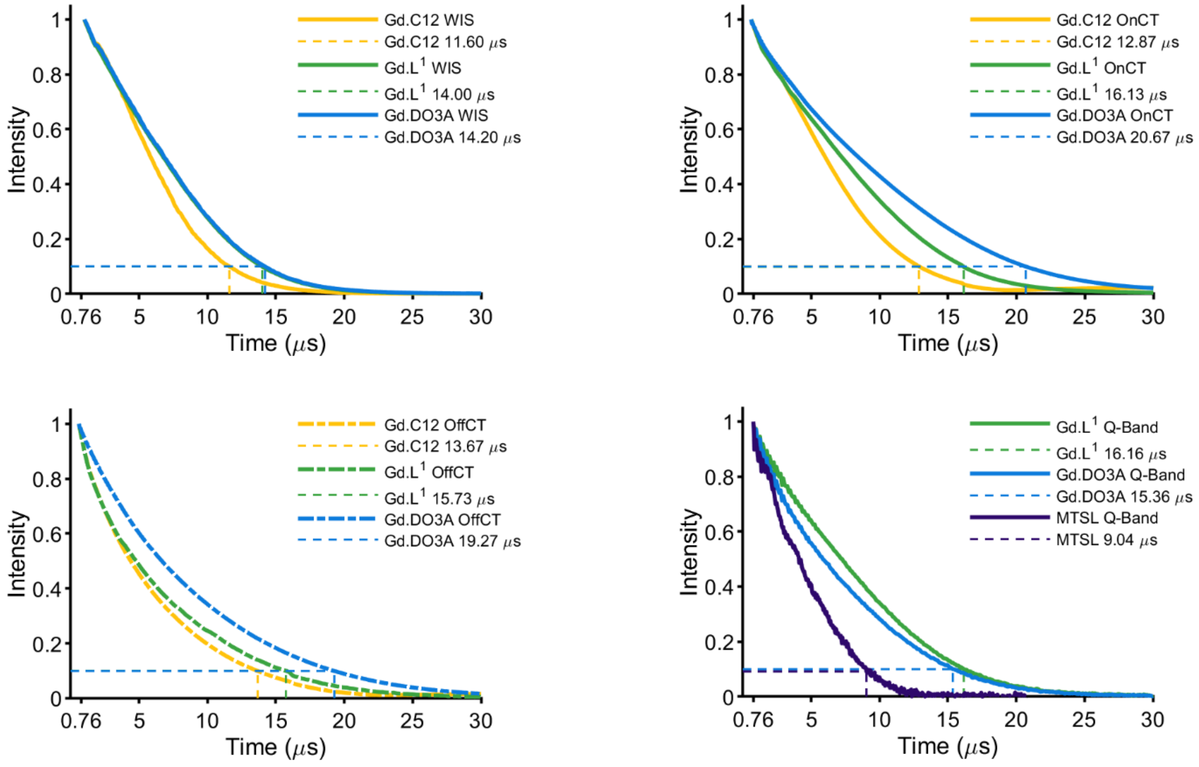

Figure S5 – Echo-detected decay curves for the Gd(III)- and MTSL-labeled CaMM13. The 10% echo remaining time is represented through a dotted line and the value registered in the legend in μs. The time axes is 2τ. Top left: WIS; Top right: HiPER on the CT; Bottom left: HiPER OffCT; Bottom right: Q-band.

Q-Q plots of the distance distributions

The following Q-Q (quartile-quartile) plot comparisons were made using the qqplot function in Matlab. The underlying DEER-derived distance distributions are those shown in Figure 3 with no error included. The more similar the distance distributions from any two data sets are, the greater the linearity of the Q-Q plot. To aid visual analysis the distance distributions were normalized to their maximum and a line from (0,0) to (1,1) has also been plotted.

The results are first shown by spin label type, as in Figure 3. The plots indicate in a visual way that for Gd.C12 the HiPER OffCT results are similar, but not the same as, the HiPER OnCT, which are again similar to the WIS results – see main paper discussion - but the HiPER OffCT and WIS results are less similar. For Gd.L^1, the HiPER OnCT, WIS and Q-band distance distributions are very similar. The HiPER OffCT are more different. For Gd.DO3A the W-band and Q-band results all give an approximately linear Q-Q plot, indicating that for this spin label the measurable distance distribution was similar across the three spectrometers and methods.

The comparison between the HiPER OffCT results in Figure 4A is then made. These Q-Q plots show that the distributions from Gd.C12 and Gd.L^1 are very similar, and both differ somewhat from Gd.DO3A. Table 2 also shows that the most probable distance is slightly shifted for Gd.DO3A compared to the Gd.C12 and Gd.L^1 results.

Finally, Gd.C12 HiPER OffCT (as a representative data set for Gd(III)-labeled CaM) is compared to MTSL. As expected, the Q-Q plot is far from linear: the distribution shown in Figure 4C also clearly shows a different most probable distance for the MTSL compared to the Gd(III) labels on the CaM.

Gd.C12

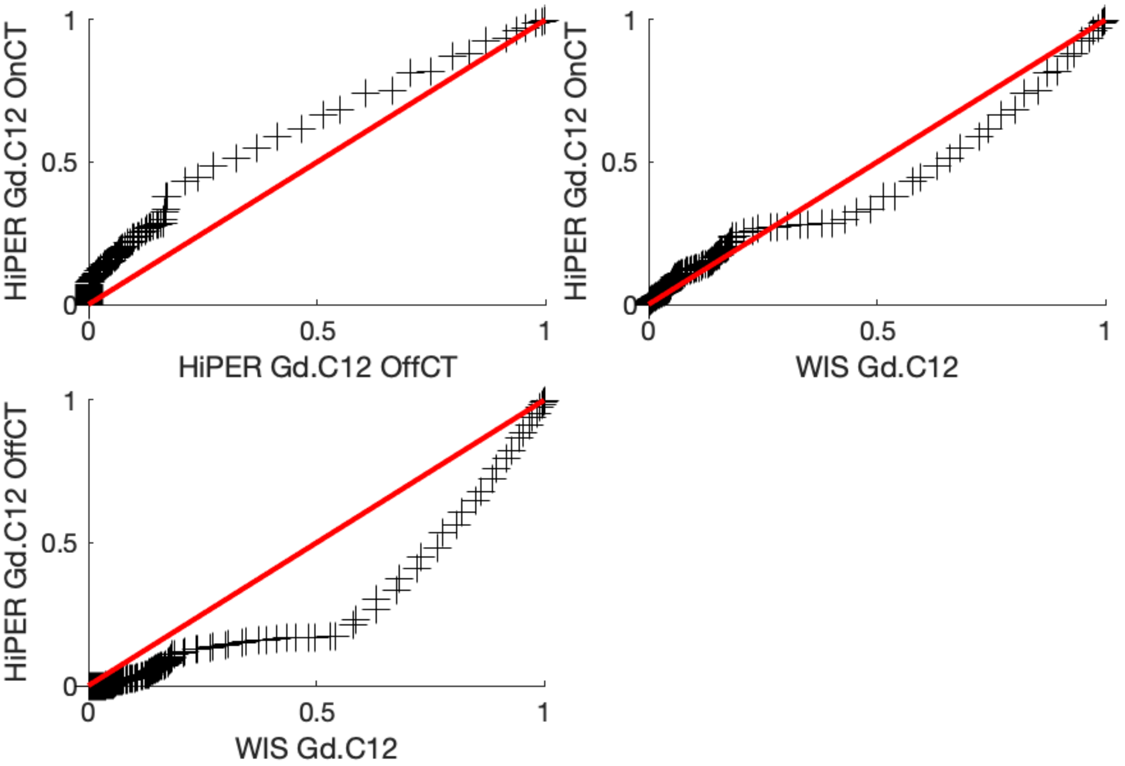
HiPER Gd.C12 OnCT
HiPER Gd.C12 OffCT
WIS Gd.C12
HiPER Gd.C12 OnCT
WIS Gd.C12
HiPER Gd.C12 OffCT
0
0.5
1

Gd.L^1

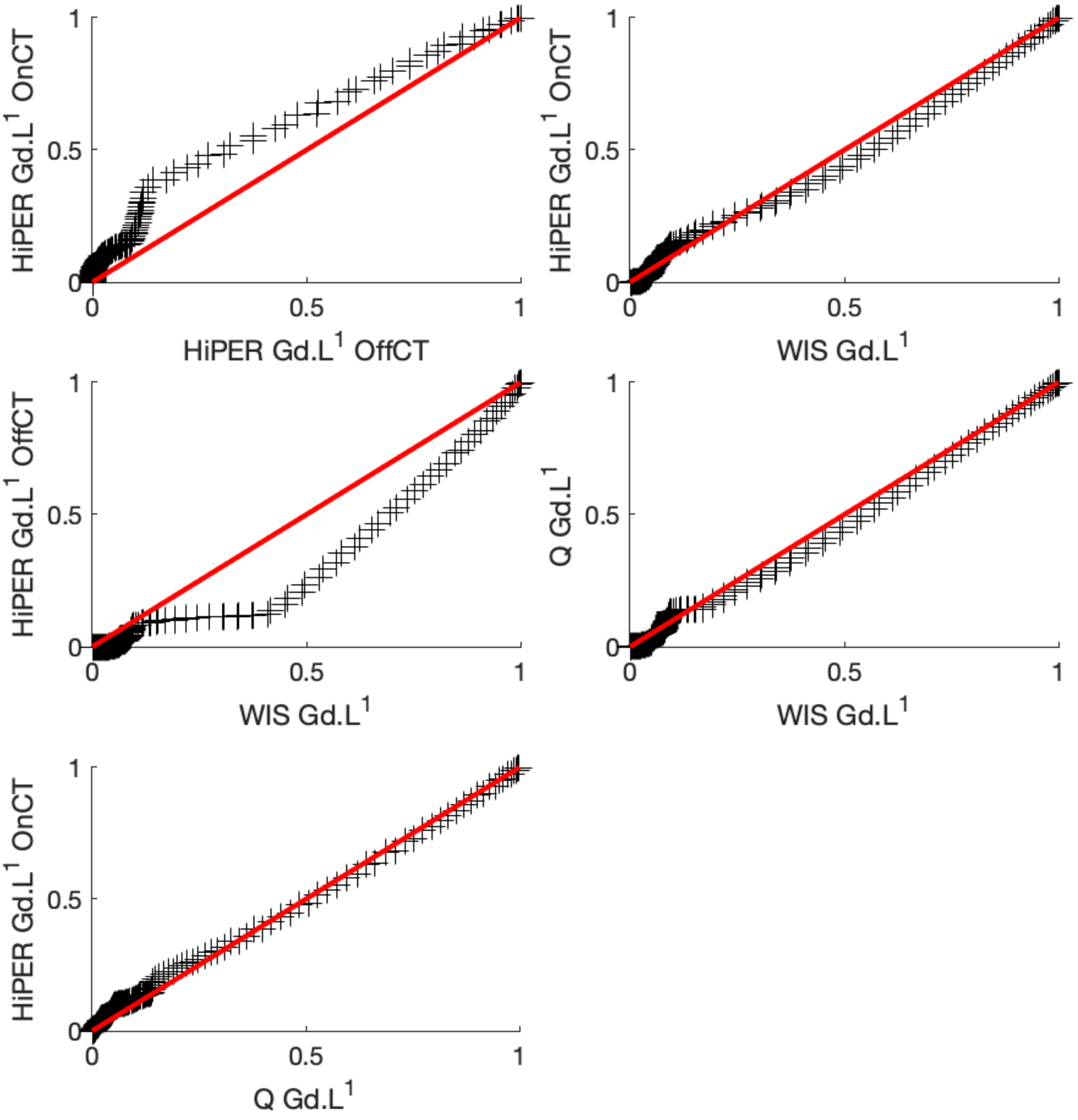

HiPER Gd.L1 OnCT
HiPER Gd.L1 OffCT
WIS Gd.L1
HiPER Gd.L1 OffCT
WIS Gd.L1
Q Gd.L1
WIS Gd.L1
HiPER Gd.L1 OnCT
Q Gd.L1
0
0.5
1

Gd.DO3A

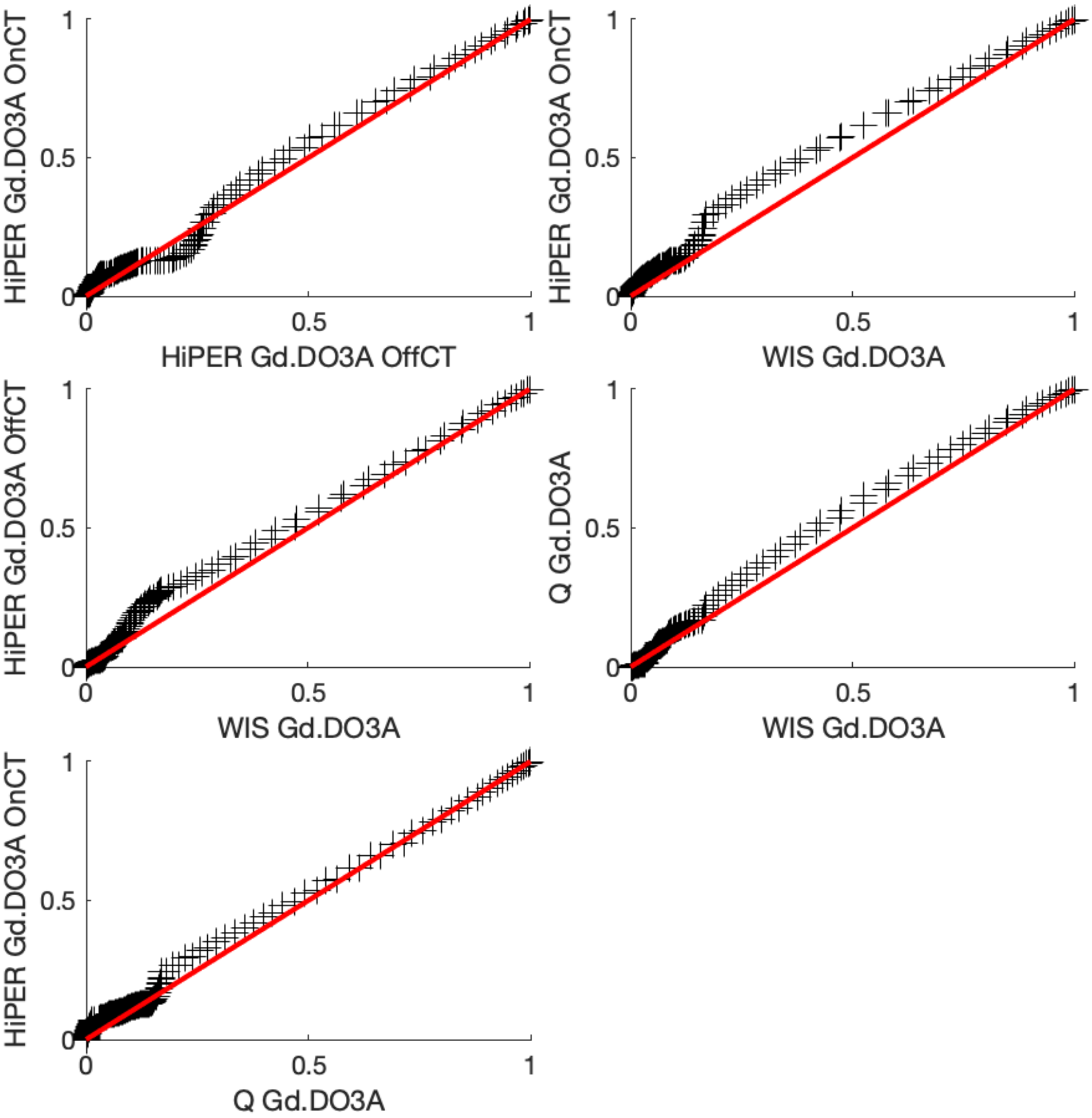

HiPER Gd.DO3A OnCT
HiPER Gd.DO3A OffCT
HiPER Gd.DO3A OnCT
WIS Gd.DO3A
HiPER Gd.DO3A OffCT
WIS Gd.DO3A
Q Gd.DO3A
WIS Gd.DO3A
HiPER Gd.DO3A OnCT
Q Gd.DO3A
0
0.5
1

HiPER OffCT

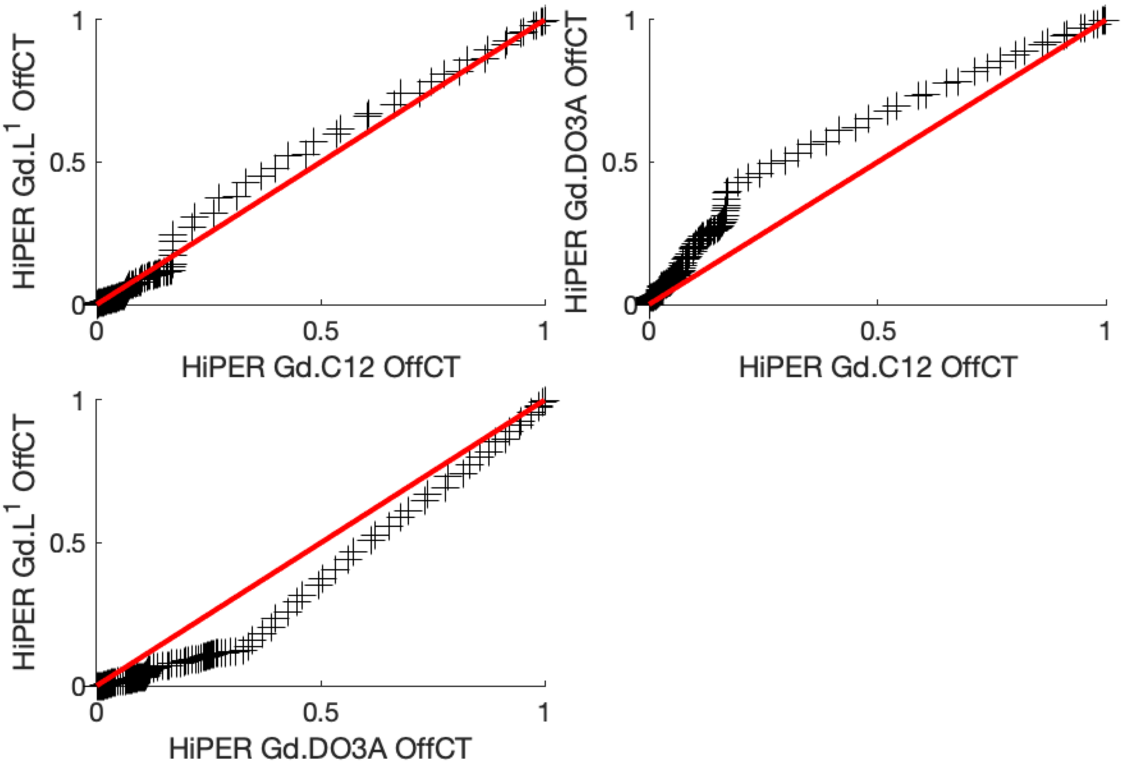

MTSL

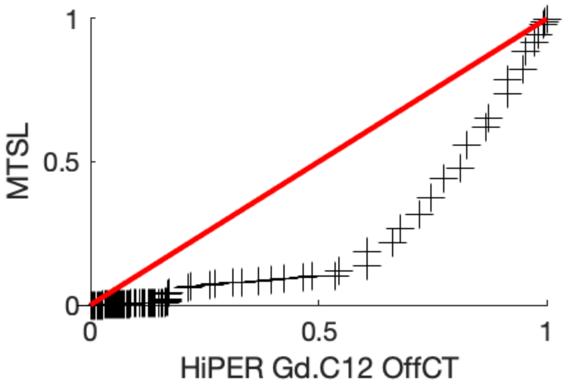

Figure S6 – Q-Q plots for the DEER-derived distance distributions presented in Figures 3 and 4.

CaMM13-Gd.DO3A HiPER results for 10 K and 6 K

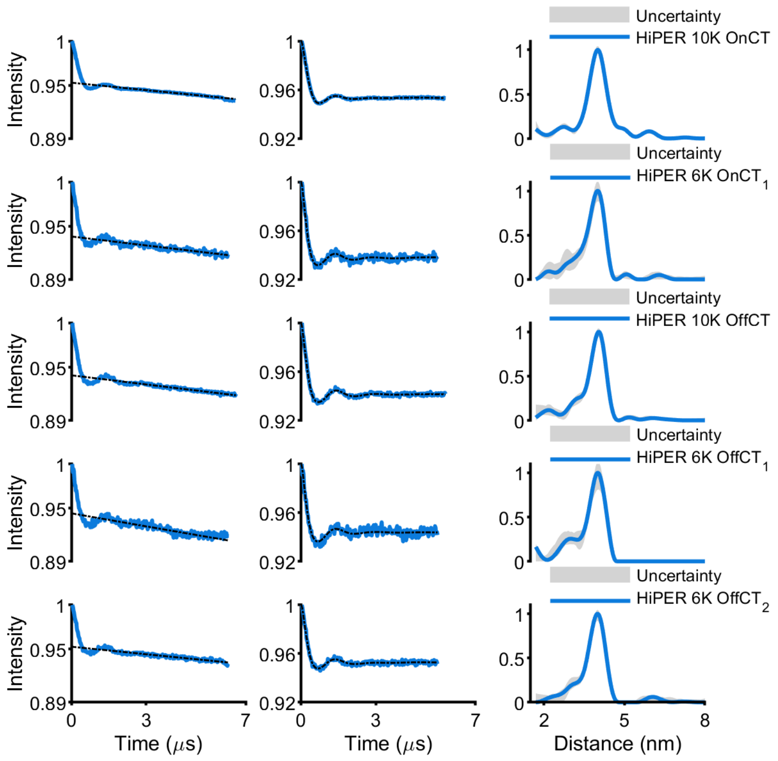

Figure S7 – HiPER DEER data and distance distributions at 10 K and 6 K for CaMM13-Gd.DO3A. Note the 6 K data is taken with fewer scans – see value for n in Table S5.

Table S5 – The experimental parameters for the four Gd.DO3A-labeled CaMM13 experiments at 10 K and 6 K (labeled as $_1$ and $_2$) on HiPER.

Gd.DO3A	HiPER OnCT	HiPER OnCT$_1$	HiPER OffCT	HiPER OffCT$_1$	HiPER OffCT$_2$
**Sample Properties**					
**Protein concentration c**	20 µM	20 µM	20 µM	20 µM	20 µM
**Volume of the sample V**	90 µL	90 µL	90 µL	90 µL	90 µL
**Glycerol**	50%	50%	50%	50%	50%
**DEER Parameters**					
**Temperature**	10 K	6 K	10 K	6 K	6 K
**No of shots**	5000	5000	5000	5000	5000
**No of scans**	100	1	20	1	4
**No of points**	351	340	351	356	341
**Phase cycling steps**	/	/	/	/	/
**Shot repetition time (SRT)**	206.17 µs	206.64 µs	207.9 µs	206.17 µs	206.73 µs
**Total measuring time**	/	/	/	/	/
**Pump frequency**	94 GHz	94.0 GHz	94.2 GHz	94.1 GHz	94.2 GHz
	100 MHz	94.5 GHz	94.5 GHz	94.5 GHz	94.5 GHz
**Pump pulse shape**	HS4	Chirp	Chirp	Chirp	Chirp
**Pump pulse total length (π)**	60 ns	120 ns	100 ns	100 ns	100 ns
**Observer frequency**	93.7 GHz	93.7 GHz	93.7 GHz	93.7 GHz	93.7 GHz
**Observer pulse length (π/2)**	HS4 30 ns	HS4 30 ns	HS4 30 ns	HS4 30 ns	HS4 30 ns
**Observer pulse length (π)**	HS4 60 ns	HS4 60 ns	HS4 60 ns	HS4 60 ns	HS4 60 ns
**Tau1**	600 ns	600 ns	600 ns	600 ns	600 ns
**Tau2**	7 µs	7 µs	7 µs	7 µs	7 µs
**Tau averages**	0	0	0	0	0
**Data Analysis**					
**Modulation depth**	4.70%	6.20%	5.90%	5.60%	4.70%
**Window size for smoothed signal**	5	5	5	5	5

**Standard deviation**	0.00031916	0.0015688	0.00084297	0.002038	0.00097455
**SNR of measurement (mod depth/Std deviation)**	147.3	39.5	70.0	27.5	48.2
**n (phase cycle*no shots*no scans)**	500000	5000	100000	5000	20000
**SNR (SNR of measurement/√n)**	0.21	0.56	0.22	0.39	0.34
**SNR per mol (SNR/(c*V)) $nmol^{-1}$**	0.12	0.31	0.12	0.22	0.19
**SNR per concentration (SNR/c) mM^{-1}**	10	28	11	19	17
**DeerAnalysis Parameters**					
**Maximum time (ns)**	6580	6280	6600	6280	6280
**Cutoff (ns)**	5780	5480	5800	5480	5480
**Zero time (ns)**	415	493	399	505	498
**Background (ns)**	1000	1400	1440	1900	840
**Tikhonov regularization parameter**	126	159	159	200	200

References.

(1) Gempf, K. L.; Butler, S. J.; Funk, A. M.; Parker, D. Direct and selective tagging of cysteine residues in peptides and proteins with 4-nitropyridyl lanthanide complexes. *Chemical Communications* **2013**, *49* (80), 9104-9106, 10.1039/C3CC45875J. DOI: 10.1039/C3CC45875J.

(2) Cruickshank, P. A. S.; Bolton, D. R.; Robertson, D. A.; Hunter, R. I.; Wylde, R. J.; Smith, G. M. A kilowatt pulsed 94 GHz electron paramagnetic resonance spectrometer with high concentration sensitivity, high instantaneous bandwidth, and low dead time. *Review of Scientific Instruments* **2009**, *80* (10). DOI: 10.1063/1.3239402 (acccessed 6/26/2024).

(3) Bahrenberg, T.; Rosenski, Y.; Carmieli, R.; Zibzener, K.; Qi, M.; Frydman, V.; Godt, A.; Goldfarb, D.; Feintuch, A. Improved sensitivity for W-band Gd(III)-Gd(III) and nitroxide-nitroxide DEER measurements with shaped pulses. *Journal of Magnetic Resonance* **2017**, *283*, 1-13. DOI: https://doi.org/10.1016/j.jmr.2017.08.003.

(4) Mentink-Vigier, F.; Collauto, A.; Feintuch, A.; Kaminker, I.; Tarle, V.; Goldfarb, D. Increasing sensitivity of pulse EPR experiments using echo train detection schemes. *Journal of Magnetic Resonance* **2013**, *236*, 117-125. DOI: https://doi.org/10.1016/j.jmr.2013.08.012.

(5) Gromov, I.; Krymov, V.; Manikandan, P.; Arieli, D.; Goldfarb, D. A W-Band Pulsed ENDOR Spectrometer: Setup and Application to Transition Metal Centers. *Journal of Magnetic Resonance* **1999**, *139* (1), 8-17. DOI: https://doi.org/10.1006/jmre.1999.1762.

(6) Epel, B.; Gromov, I.; Stoll, S.; Schweiger, A.; Goldfarb, D. Spectrometer manager: A versatile control software for pulse EPR spectrometers. *Concepts in Magnetic Resonance Part B: Magnetic Resonance Engineering* **2005**, *26B*, 36-45. DOI: 10.1002/cmr.b.20037.

(7) Pannier, M.; Veit, S.; Godt, A.; Jeschke, G.; Spiess, H. W. Dead-time free measurement of dipole-dipole interactions between electron spins. *J Magn Reson* **2000**, *142* (2), 331-340. DOI: 10.1006/jmre.1999.1944 From NLM Medline.

(8) Stoll, S.; Schweiger, A. EasySpin, a comprehensive software package for spectral simulation and analysis in EPR. *Journal of Magnetic Resonance* **2006**, *178* (1), 42-55. DOI: https://doi.org/10.1016/j.jmr.2005.08.013.
